# Correction: Microbial phenotypic heterogeneity in response to a metabolic toxin: Continuous, dynamically shifting distribution of formaldehyde tolerance in *Methylobacterium extorquens* populations

**DOI:** 10.1371/journal.pgen.1010714

**Published:** 2023-04-05

**Authors:** Jessica A. Lee, Siavash Riazi, Shahla Nemati, Jannell V. Bazurto, Andreas E. Vasdekis, Benjamin J. Ridenhour, Christopher H. Remien, Christopher J. Marx

The S1 Data file in the list of Supporting Information is incorrect. Please view the correct S1 Data file below.

## Supporting information

S1 Data Excel file with original data shown in each of Figs 1, 2, 4, 5, 6 and 7. (XLSX)
