# Supplementary material for: Microbial phenotypic heterogeneity in response to a metabolic toxin: Continuous, dynamically shifting distribution of formaldehyde tolerance in Methylobacterium extorquens populations
Source: PLoS Genet. 2019 Nov 11;15(11):e1008458. doi: 10.1371/journal.pgen.1008458 (PMC6858071; doi:10.1371/journal.pgen.1008458)
Supplement: S1 Fig — (PDF) [file pgen.1008458.s001.pdf]

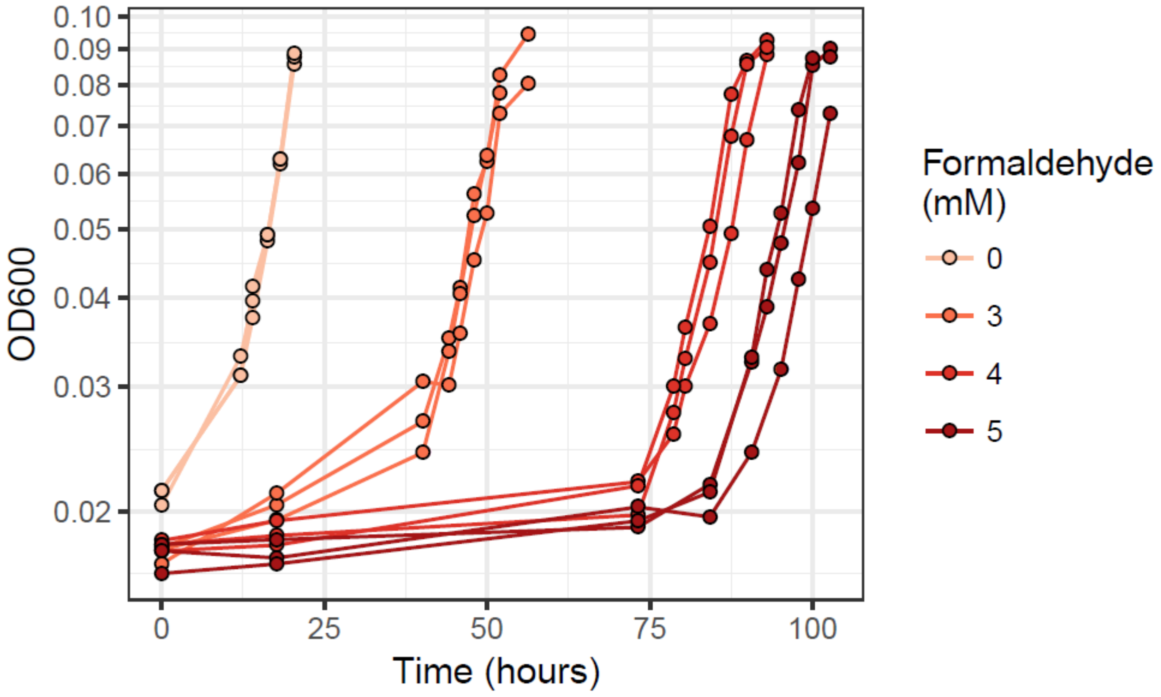

Figure S1. Formaldehyde concentrations of ≤5 mM allow growth of *M. extorquens* at a normal rate, but only after a period of lag; higher concentrations lead to longer lag times.
Isogenic populations of WT *M. extorquens* were inoculated into culture flasks with fresh MPIPES medium with 15 mM methanol and the indicated concentration of formaldehyde; samples were removed regularly for measurement of optical density at 600 nm. Each line represents one biological replicate.
