# Supplementary material for: Microbial phenotypic heterogeneity in response to a metabolic toxin: Continuous, dynamically shifting distribution of formaldehyde tolerance in Methylobacterium extorquens populations
Source: PLoS Genet. 2019 Nov 11;15(11):e1008458. doi: 10.1371/journal.pgen.1008458 (PMC6858071; doi:10.1371/journal.pgen.1008458)
Supplement: S2 Fig — (PDF) [file pgen.1008458.s002.pdf]

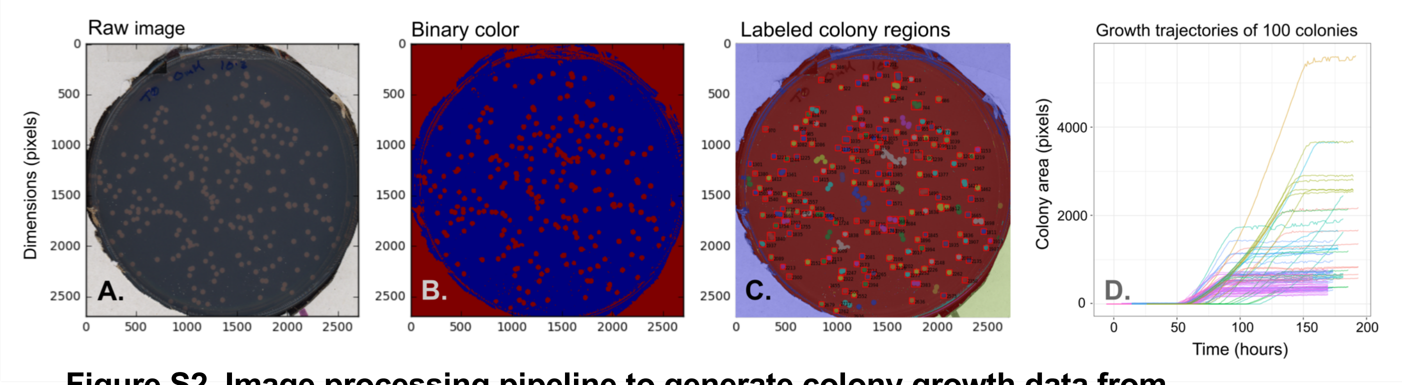

Figure S2. Image processing pipeline to generate colony growth data from formaldehyde-exposed cultures.

A) Color images of culture plates incubating on a flatbed photo scanner were captured once per hour; images were processed in Python 3.5.6 using a custom script employing sci-kit image v.0.12.1.

B) Each scanner bed held 6 plates; color processing was carried out for all 6 plates in an image together. The red channel only was used; the image was converted to binary color using the theshold_otsu tool to determine the threshold between light (colony) and dark (background plate) pixels, shown here as red and blue respectively.

C) For each plate, the final image was used to identify pixels belonging to each colony, using the label tool to designate colonies as individual regions. To eliminate non-colony objects and groups of multiple colonies that had grown together, a custom script was used to eliminate regions that did not meet the following criteria: 1) the region was between 40 and 10,000 pixels in size; 2) the ratio between the two dimensions of the bounding box was between 0.6 and 1.4 (approximately square), and 3) the region area occupied at least 60% of its bounding box (mostly convex). After the automatic criteria were employed, images of labeled regions were then also inspected visually, and any regions that were not single colonies were eliminated manually from downstream analysis.

D) Once the regions belonging to each colony were identified in the final image, all the images of the time series were converted to binary color and the number of bright pixels within each colony's region (the colony area) was enumerated at each timepoint. These data were imported into R v.3.4.3 for further analysis. Timepoints were adjusted to account for the fact that plates had begun growing at different times (as each population had been exposed to formaldehyde for a different length of time prior to plating). Any objects that had an area of >100 pixels at the first timepoint, or that never reached an area of 100 pixels by the final timepoint, were identified as non-colony objects and omitted from further analysis. Panel D shows growth trajectories of 100 randomly chosen colonies as an example. Colony appearance times were measured as the first timepoint at which a colony reached an area of 100 pixels or greater. Growth rates were calculated by fitting a linear relationship between time (in hours) and the binary logarithm of colony area (in pixels) using the lm function. Only growth rate estimates with an adjusted R^2 of >0.95 were retained.
