# Supplementary material for: Microbial phenotypic heterogeneity in response to a metabolic toxin: Continuous, dynamically shifting distribution of formaldehyde tolerance in Methylobacterium extorquens populations
Source: PLoS Genet. 2019 Nov 11;15(11):e1008458. doi: 10.1371/journal.pgen.1008458 (PMC6858071; doi:10.1371/journal.pgen.1008458)
Supplement: S3 Fig — (PDF) [file pgen.1008458.s003.pdf]

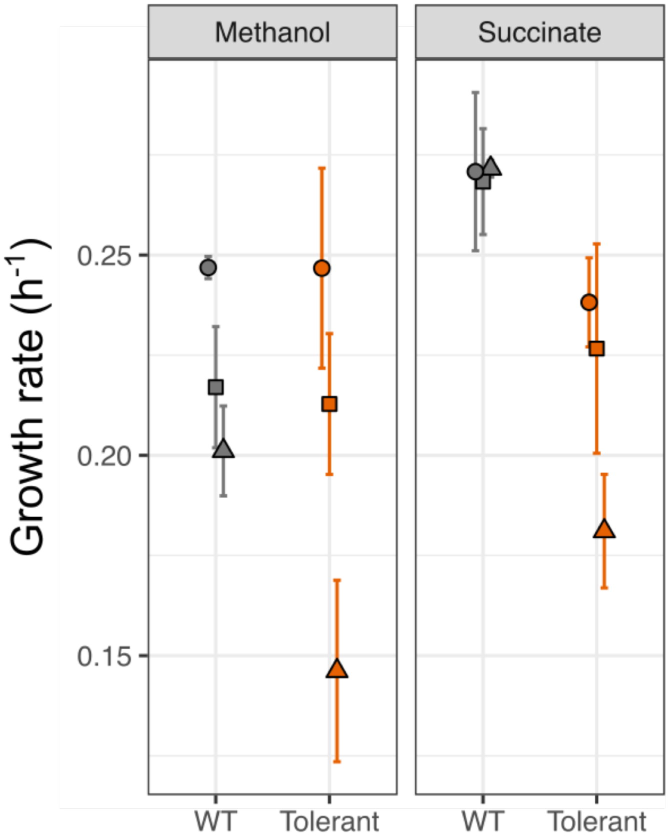


**Figure S3**. **Formaldehyde tolerance may be associated with lower fitness on a multicarbon substrate.**
Three *M. extorquens* populations with elevated tolerance were generated by selecting for tolerant cells via a 4 mM formaldehyde exposure experiment. These ("Tolerant") populations were then compared to three non-selected ("WT") populations during growth on medium without formaldehyde, with either methanol (left) or succinate (right) as the sole carbon source. Error bars represent the standard deviation of three replicate incubations of each population. Symbols represent the inocula from which the populations originated (e.g., all squares came from the same overnight culture prior to selection). On succinate only, naive *M. extorquens* populations grow marginally faster than tolerant populations, though the difference was not statistically significant (ANOVA: $F$=2.617, $p$=0.123 for the model; $p$=0.940 for the planned contrast between the two populations on methanol and $p$=0.136 on succinate).
