# Supplementary material for: Microbial phenotypic heterogeneity in response to a metabolic toxin: Continuous, dynamically shifting distribution of formaldehyde tolerance in Methylobacterium extorquens populations
Source: PLoS Genet. 2019 Nov 11;15(11):e1008458. doi: 10.1371/journal.pgen.1008458 (PMC6858071; doi:10.1371/journal.pgen.1008458)
Supplement: S4 Fig — (PDF) [file pgen.1008458.s004.pdf]

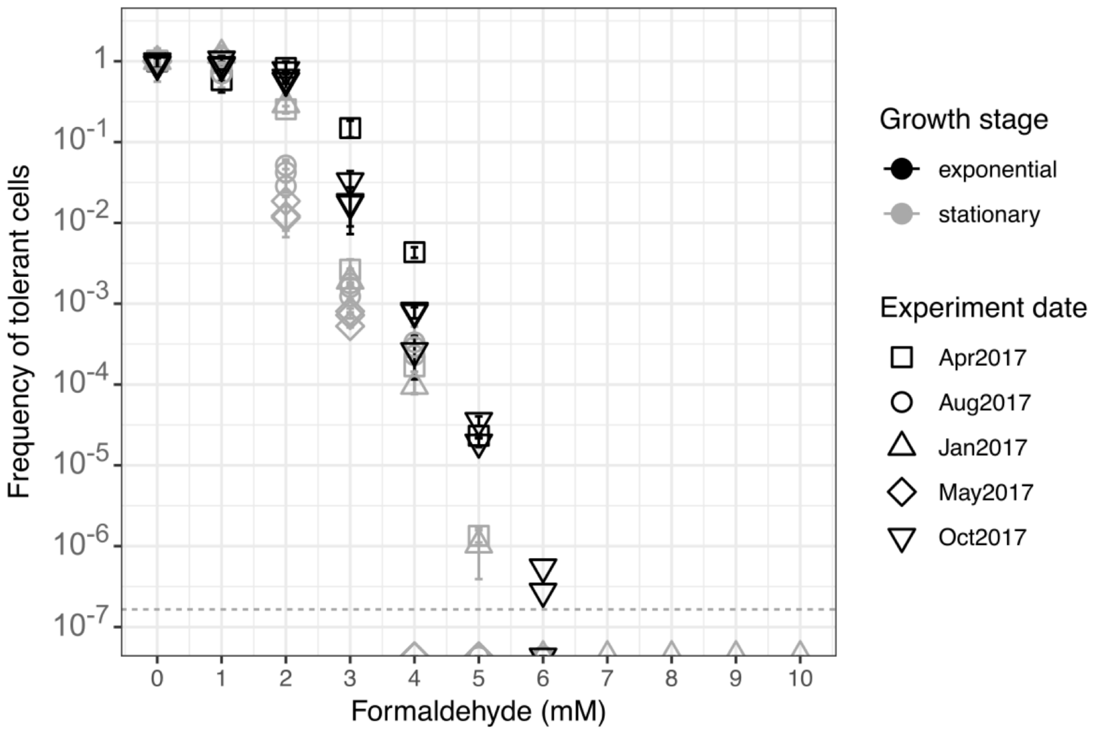

Figure S4. Formaldehyde tolerance distributions in *Methylobacterium* populations are robust across experimental replicates, but vary depending on growth conditions. Formaldehyde tolerance distributions were measured in isogenic *M. extorquens* populations grown on methanol medium and not previously exposed to formaldehyde, by plating onto agar methanol medium containing formaldehyde at the indicated concentrations. The frequency of tolerant cells is expressed as the ratio of the number of colonies observed on formaldehyde medium at the specified concentration to the number of colonies on formaldehyde-free (0 mM) medium. Shown here are results from several replicate experiments at different times; the average values from all replicates are shown in Fig. 6. Error bars denote the standard deviation of 3 replicate platings, and detection limit is indicated by the dashed horizontal line. In general, stationary-phase populations have lower overall tolerance than exponential-phase (actively growing) populations.
