# Supplementary material for: Microbial phenotypic heterogeneity in response to a metabolic toxin: Continuous, dynamically shifting distribution of formaldehyde tolerance in Methylobacterium extorquens populations
Source: PLoS Genet. 2019 Nov 11;15(11):e1008458. doi: 10.1371/journal.pgen.1008458 (PMC6858071; doi:10.1371/journal.pgen.1008458)
Supplement: S5 Fig — (PDF) [file pgen.1008458.s005.pdf]

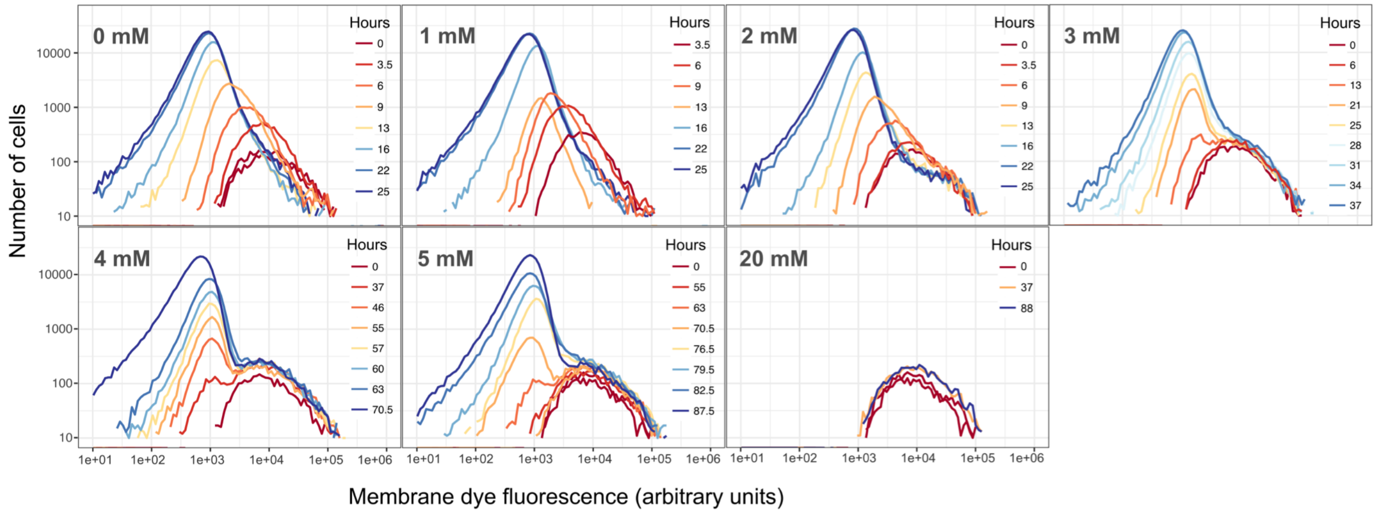

Figure S5. Cell proliferation assays support the hypothesis that growth of *M. extorquens* in the presence of formaldehyde is due to a small subpopulation of tolerant cells, and that the abundance of tolerant cells decreases with increasing formaldehyde.

(See also Fig. 3.) A naive culture of *M. extorquens* was stained using a fluorescent membrane dye and divided among culture flasks with different concentrations of formaldehyde; samples were periodically removed, fixed, and then analyzed by flow cytometry. Panels show formaldehyde treatment concentration; each line is the outline of a histogram of per-cell fluorescence measurements from one timepoint. For each sample, an equal volume was analyzed: higher cell counts indicate a higher density of cells in the culture. Per-cell membrane fluorescence decreases with growth, as cell division results in the dilution of membrane dye between daughter cells. Non-growing cells show no change in per-cell fluorescence. At 0 mM formaldehyde, all cells grow; at 20 mM, no cells grow; at intermediate concentrations, populations contain some growing and some non-growing cells.
