# Supplementary material for: Microbial phenotypic heterogeneity in response to a metabolic toxin: Continuous, dynamically shifting distribution of formaldehyde tolerance in Methylobacterium extorquens populations
Source: PLoS Genet. 2019 Nov 11;15(11):e1008458. doi: 10.1371/journal.pgen.1008458 (PMC6858071; doi:10.1371/journal.pgen.1008458)
Supplement: S6 Fig — (PDF) [file pgen.1008458.s006.pdf]

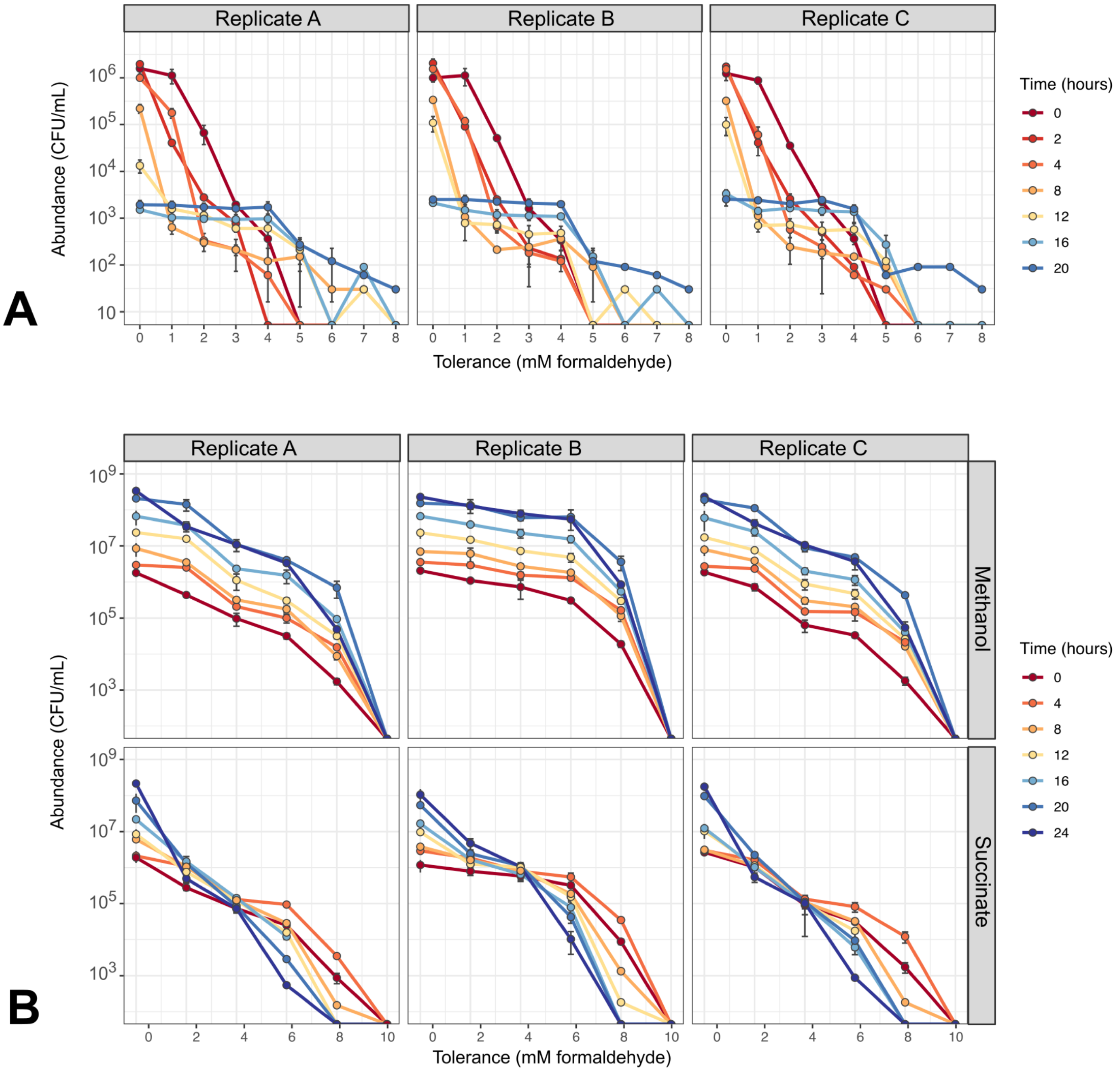

Figure S6. The distribution of formaldehyde tolerance within an *M. extorquens* population changes over time depending on growth conditions.
A) exposure to 4 mM formaldehyde selecting for tolerant cells; and B) regrowth of a tolerant population on formaldehyde-free medium (top row: methanol as the growth substrate; bottom row: succinate). Each column represents a separate biological replicate; error bars denote the standard deviation of three replicate platings for colony counts. Replicate B alone is shown in Fig. 7.
