# Supplementary material for: Microbial phenotypic heterogeneity in response to a metabolic toxin: Continuous, dynamically shifting distribution of formaldehyde tolerance in Methylobacterium extorquens populations
Source: PLoS Genet. 2019 Nov 11;15(11):e1008458. doi: 10.1371/journal.pgen.1008458 (PMC6858071; doi:10.1371/journal.pgen.1008458)
Supplement: S7 Fig — (PDF) [file pgen.1008458.s007.pdf]

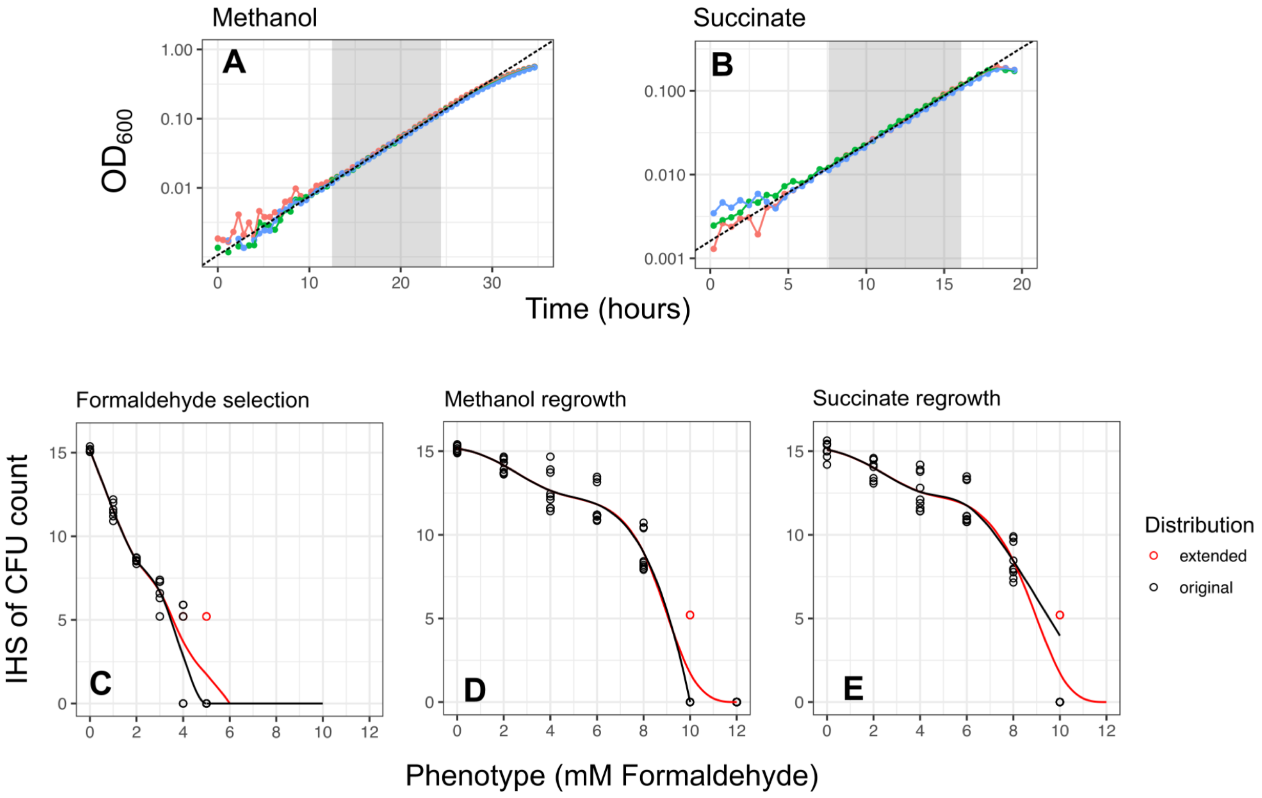

Figure S7. Estimation of growth rates and initial conditions for use in the mathematical model.

A) and B) Growth rates on methanol and succinate (r_m, r_s respectively) were estimated by linear regression using data from *M. extorquens* cultures grown without formaldehyde on each of the two growth substrates. Gray shaded region indicates portion of growth curve used; dashed lines show exponential fit. OD_{600}: optical density at 600 nm. C-E) Initial conditions used for each of the three model scenarios. Points show experimental data (transformed by inverse hyperbolic sine) and line shows spline fit. Black points and line indicate original measured distribution; red points and lines indicate distribution extended to correct for undetected high-tolerance cells possibly present at abundances below the experimental detection limit. The extended distributions were used for the model.
