# Supplementary material for: Microbial phenotypic heterogeneity in response to a metabolic toxin: Continuous, dynamically shifting distribution of formaldehyde tolerance in Methylobacterium extorquens populations
Source: PLoS Genet. 2019 Nov 11;15(11):e1008458. doi: 10.1371/journal.pgen.1008458 (PMC6858071; doi:10.1371/journal.pgen.1008458)
Supplement: S8 Fig — (PDF) [file pgen.1008458.s008.pdf]

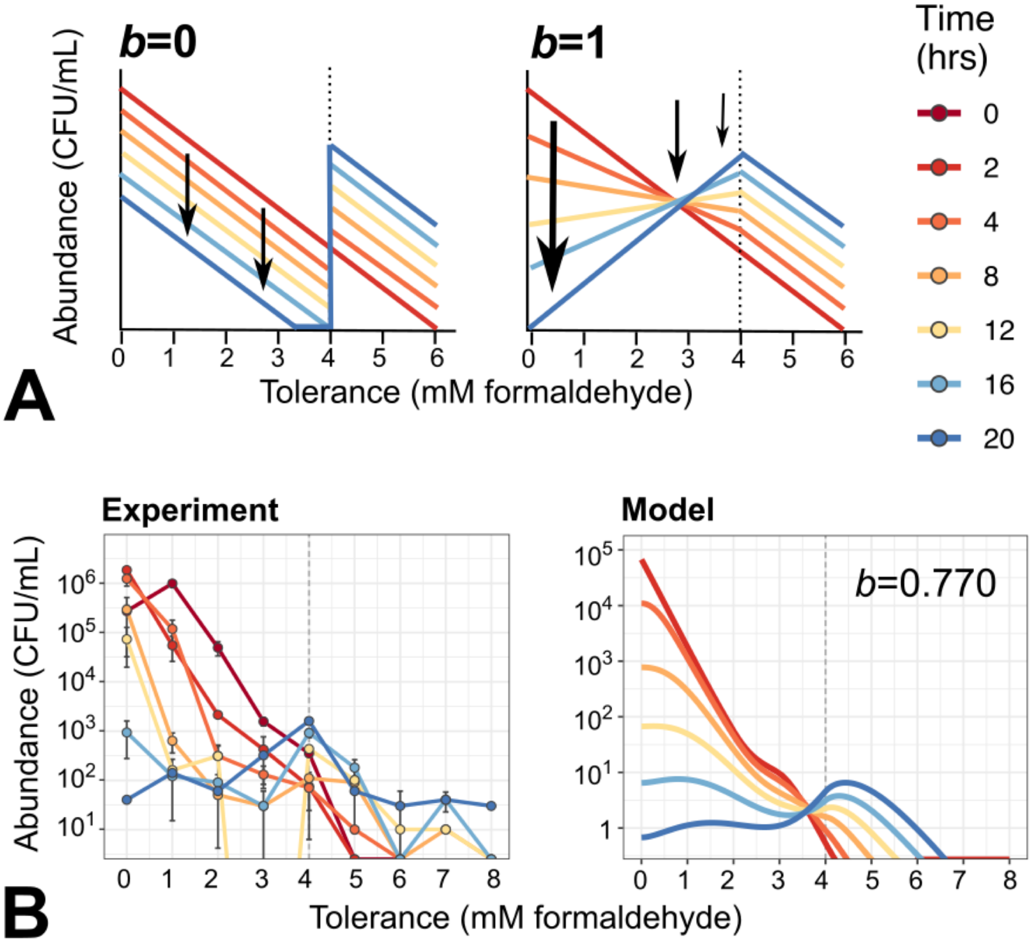

Figure S8. The parameter *b* (dependence of death rate on formaldehyde tolerance) determines the shape of the population's phenotypic tolerance distribution after exposure to formaldehyde.

Distributions are displayed in the non-cumulative form (see Methods for details). A) Schematic showing theoretical population shifts at exposure to 4 mM formaldehyde. When *b*=0, all cells die at the same rate regardless of their formaldehyde tolerance, as long as their tolerance level is lower than the formaldehyde concentration. In this case, formaldehyde exposure results in a tolerance distribution with two peaks: at *x*=0 and *x*=*F*. When *b*=1, death rate is proportional to tolerance, such that cells with higher *x* die more slowly. This results in a tolerance distribution with one peak: at *x*=*F*. B) Results of (left) experiment and (right) model simulation: distribution falls partly between the two extremes. Note that the experimental results shown here are transformed into the non-cumulative form for comparison, as described in the Methods; and CFU abundances in model appear lower than in experiment because the continuous results have not been binned into 1-mM increments for this display.
