# Supplementary material for: Microbial phenotypic heterogeneity in response to a metabolic toxin: Continuous, dynamically shifting distribution of formaldehyde tolerance in Methylobacterium extorquens populations
Source: PLoS Genet. 2019 Nov 11;15(11):e1008458. doi: 10.1371/journal.pgen.1008458 (PMC6858071; doi:10.1371/journal.pgen.1008458)
Supplement: S9 Fig — (PDF) [file pgen.1008458.s009.pdf]

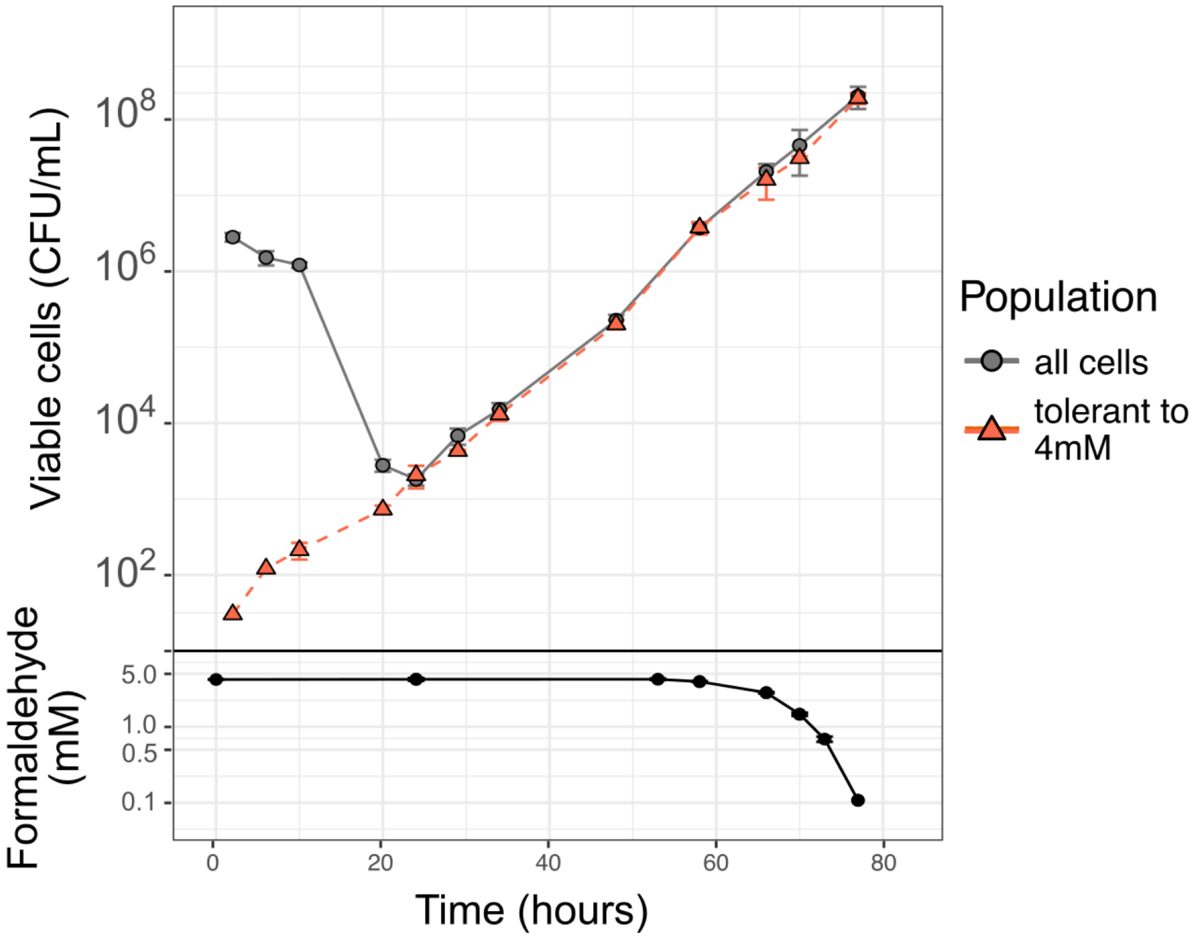

Figure S9. Cells expressing mCherry show the same formaldehyde tolerance heterogeneity as wild-type *M. extorquens* cells.
To facilitate identification of cells by flow cytometry in the cell proliferation assay, we used CM3839, a strain of *M. extorquens* PA1 engineered for the constitutive expression of the red fluorescent protein mCherry [64]. To ensure that the red-fluorescent strain showed the same phenotypic heterogeneity in formaldehyde tolerance as the wild-type strain (CM2730), we conducted a 4 mM formaldehyde exposure experiment with CM3839. Just as with CM2730 (Fig. 2), CM3839 populations contain a small subpopulation of cells that are tolerant to 4 mM formaldehyde, and that grow at a normal rate when the population is exposed to formaldehyde at that concentration. Error bars denote the standard deviation of three replicate measurements (for the formaldehyde measurements, error bars fall within the area of the symbols).
