# Supplementary material for: Microbial phenotypic heterogeneity in response to a metabolic toxin: Continuous, dynamically shifting distribution of formaldehyde tolerance in Methylobacterium extorquens populations
Source: PLoS Genet. 2019 Nov 11;15(11):e1008458. doi: 10.1371/journal.pgen.1008458 (PMC6858071; doi:10.1371/journal.pgen.1008458)
Supplement: S10 Fig — (PDF) [file pgen.1008458.s010.pdf]

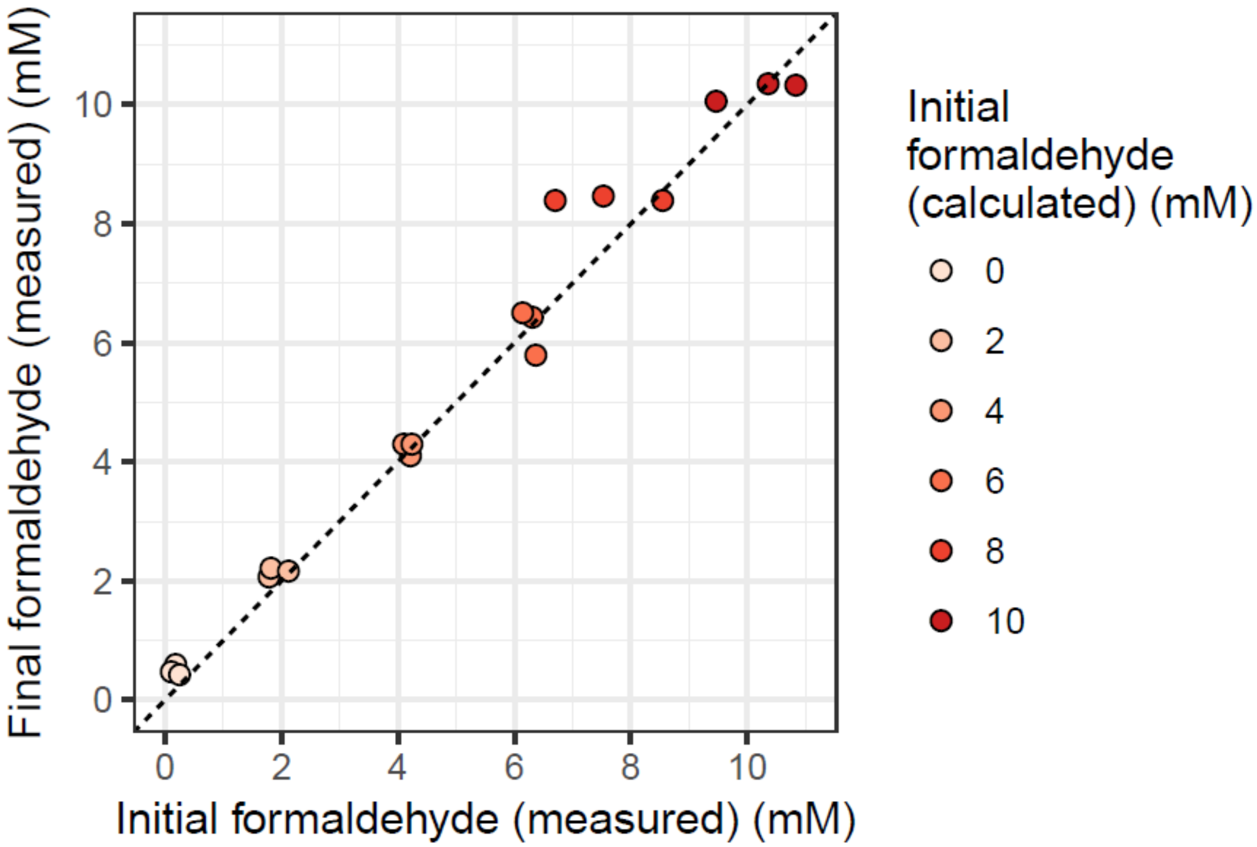

Figure S10. Formaldehyde concentrations in agar growth medium are stable over time and reflective of similar concentrations in liquid medium.
MPIPES-methanol-agar culture plates were made with the indicated concentration of formaldehyde. A small amount of agar (~0.1 g) was excised from the plate, melted, diluted 1:10 in MPIPES medium, and assayed for formaldehyde as described in Methods. Each plate was assayed in triplicate. Plates were then incubated for 3 days, stored together in the same bag, at 30ºC, and assayed again. No significant change in concentration was detected in any of the plates.
