# Supplementary material for: Microbial phenotypic heterogeneity in response to a metabolic toxin: Continuous, dynamically shifting distribution of formaldehyde tolerance in Methylobacterium extorquens populations
Source: PLoS Genet. 2019 Nov 11;15(11):e1008458. doi: 10.1371/journal.pgen.1008458 (PMC6858071; doi:10.1371/journal.pgen.1008458)
Supplement: S11 Fig — (PDF) [file pgen.1008458.s011.pdf]

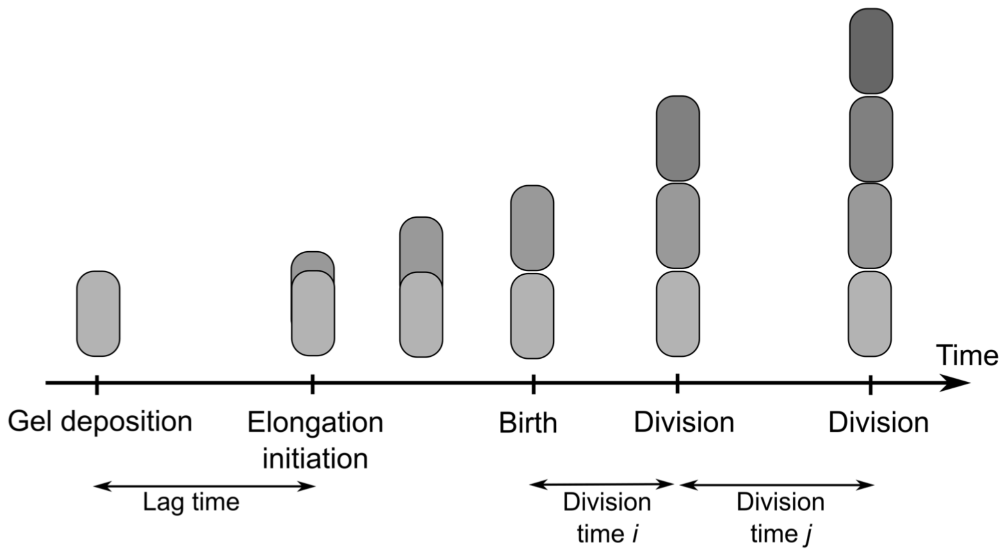

Figure S11. Time-lapse microscopy: cell segmentation and tracking.
Colony lag times and cell division times were measured as shown above: “lag time” refers to the time between deposition of cells in the gel and the beginning of cell elongation; there is only 1 lag time for each microcolony. “Division time” refers to the full time period between the formation of a cell and the formation of its daughter cell; for each microcolony, many division times were observed.
