# Supplementary material for: Microbial phenotypic heterogeneity in response to a metabolic toxin: Continuous, dynamically shifting distribution of formaldehyde tolerance in Methylobacterium extorquens populations
Source: PLoS Genet. 2019 Nov 11;15(11):e1008458. doi: 10.1371/journal.pgen.1008458 (PMC6858071; doi:10.1371/journal.pgen.1008458)
Supplement: S12 Fig — (PDF) [file pgen.1008458.s012.pdf]

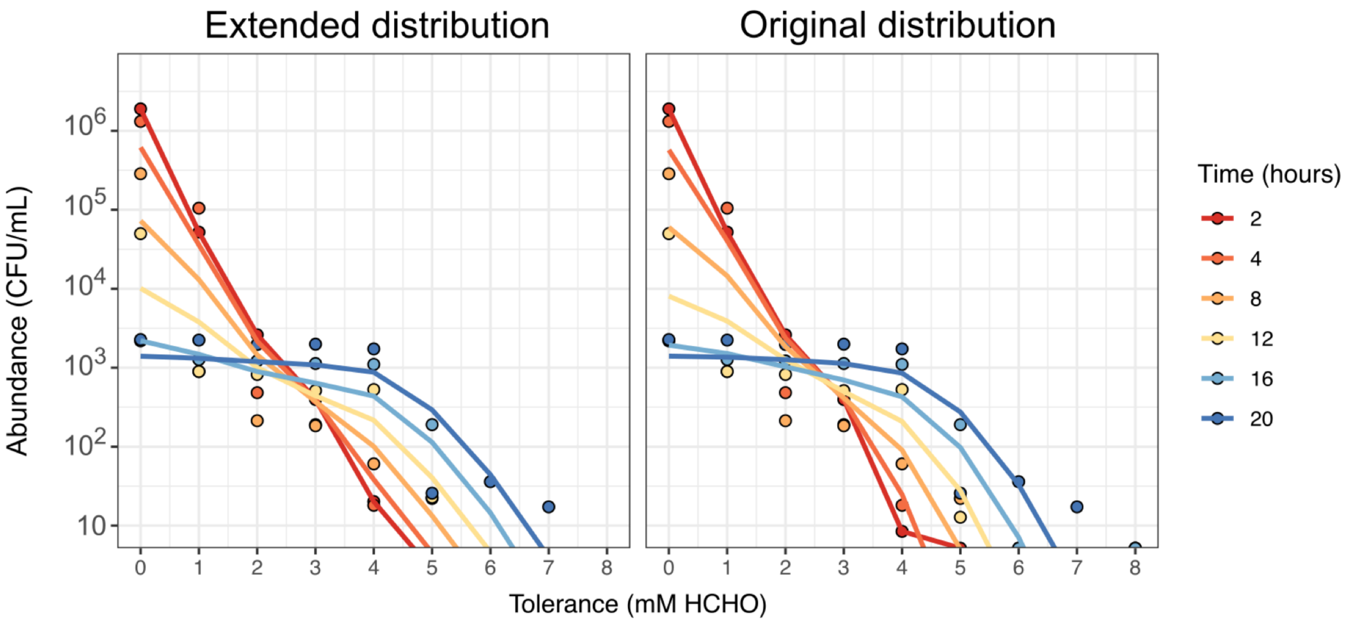

Figure S12. Models using extended and original tolerance distributions perform similarly.
Both panels show results of model simulation of population shifts during 4 mM formaldehyde selection experiment. Points: experimental data used for fitting model (averages of all replicates). Lines: model output, binned at 1 mM increments and summed to form cumulative distributions, for comparison with experimental data. Left panel: dataset in which extra CFU counts have been added at a few high-tolerance phenotype levels according to the rules described in the Methods, in order to correct for cells potentially present at abundances below the limit of detection (as in Fig. 9; included here for comparison). Right panel: dataset using only the experimentally measured counts, without extension.
