# Supplementary material for: Microbial phenotypic heterogeneity in response to a metabolic toxin: Continuous, dynamically shifting distribution of formaldehyde tolerance in Methylobacterium extorquens populations
Source: PLoS Genet. 2019 Nov 11;15(11):e1008458. doi: 10.1371/journal.pgen.1008458 (PMC6858071; doi:10.1371/journal.pgen.1008458)
Supplement: S1 Table — (PDF) [file pgen.1008458.s013.pdf]

Table S1. Tolerant subpopulation shows no significant difference in sensitivity to antibiotics or hydrogen peroxide. *M. extorquens* populations selected for tolerance via a 4 mM formaldehyde exposure experiment ("tolerant") and non-selected populations ("WT") were tested for sensitivity to H_2O_2 and antibiotics using a disk susceptibility test. Stressors were provided in the amount listed; test disks were purchased from Fisher Technical Company (Erythromycin, Kanamycin) or BD BBL Sensi-Disc (all others). Data shown here are from two experiments: an initial screening experiment conducted with no replicates, and a second experiment in triplicate.

First experiment, no replicates.			
**Stress**	**Population**	**Replicate**	**Diameter (cm)**
blank disk	WT	A	0
	tolerant	A	0
Rifampicin, 5 mg	WT	A	35
	tolerant	A	36
Vancomycin, 30 mg	WT	A	0
	tolerant	A	0
Cefoxitin, 30 mg	WT	A	0
	tolerant	A	0
Novobiocin, 5 mg	WT	A	20
	tolerant	A	23
Nalidixic Acid, 30 mg	WT	A	0
	tolerant	A	0
Erythromycin, 15 mg	WT	A	0
	tolerant	A	0
Colistin, 10 mg	WT	A	0
	tolerant	A	0
Kanamycin, 30 mg	WT	A	30
	tolerant	A	32
Gentamicin, 10 mg	WT	A	17
	tolerant	A	13
H_2O_2, 5 µL of 30% solution	WT	A	43
	tolerant	A	60
Ciprofloxacin, 10 mg	WT	A	0
	tolerant	A	8
Second experiment, triplicates.			
**Stress**	**Population**	**Replicate**	**Diameter (cm)**
H_2O_2, 3 µL of 30% solution	WT	A	53
		B	55
		C	55
	tolerant	A	60
		B	60
		C	55
Gentamicin, 10 mg	WT	A	20
		B	17
		C	18
	tolerant	A	20
		B	18
		C	18
Kanamycin, 30 mg	WT	A	26
		B	25
		C	27
	tolerant	A	30
		B	28
		C	28
Ciprofloxacin, 10 mg	WT	A	16
		B	15
		C	17
	tolerant	A	16
		B	16
		C	16
