# Supplementary material for: Microbial phenotypic heterogeneity in response to a metabolic toxin: Continuous, dynamically shifting distribution of formaldehyde tolerance in Methylobacterium extorquens populations
Source: PLoS Genet. 2019 Nov 11;15(11):e1008458. doi: 10.1371/journal.pgen.1008458 (PMC6858071; doi:10.1371/journal.pgen.1008458)
Supplement: S2 Table — (PDF) [file pgen.1008458.s014.pdf]

Table S2. Results of model selection using original data set for fitting (distribution not extended to account for experimental limit of detection).
See Table 1 for comparison. For the likelihood ratio test, the name of the model used as the null, as well as the χ^2 value and *p*-value, are given. Gray shading: the best-supported model. Pseudo-R^2 value for that model: 0.970.

Model	***Experimental scenario***		***Parameters***				***Likelihood Ratio Test***		
	**Condition**	**Substrate**	***α***	***b***	***v***	***D***	**null model**	χ^2	***p***
**F1**	selection	Methanol + Formaldehyde	0.141	n/a	n/a	n/a	n/a	n/a	n/a
**F2a**	selection	Methanol + Formaldehyde	0.186	0.925	n/a	n/a	F1	5.710	0.017
**F2b**	selection	Methanol + Formaldehyde	0.166	n/a	-0.077	n/a	F1	40.026	<0.001
**F2c**	selection	Methanol + Formaldehyde	0.158	n/a	n/a	0.041	F1	40.853	<0.001
**F3a**	selection	Methanol + Formaldehyde	0.214	0.809	n/a	0.027	F2b	9.379	0.002
**F3b**	selection	Methanol + Formaldehyde	0.163	n/a	-0.047	0.017	F2b	1.647	0.199
**F4**	selection	Methanol + Formaldehyde	0.220	0.929	0.051	0.044	F3a	0.752	0.386
