# Supplementary material for: Microbial phenotypic heterogeneity in response to a metabolic toxin: Continuous, dynamically shifting distribution of formaldehyde tolerance in Methylobacterium extorquens populations
Source: PLoS Genet. 2019 Nov 11;15(11):e1008458. doi: 10.1371/journal.pgen.1008458 (PMC6858071; doi:10.1371/journal.pgen.1008458)
Supplement: S1 File — (HTML) [file pgen.1008458.s015.html]

Modeling phenotypic switching in Methylobacterium extorquens


# Modeling phenotypic switching in *Methylobacterium extorquens*

- The PDE Model
  - Code for the PDE
  - Maximum Likelihood Fitting
- Growth on Methanol and Formaldehyde
  - The 4mM HCHO Data and Initial Conditions
  - Parameter Estimation for the 4mM Growth Experiment
  - Visualizing the Fitted Model
  - Using the “Extended” Initial Condition
  - Comparing The Parameters
    - Plots of the Extended IC Solution
- Growth in the Absence of Formaldehyde
  - Growth on Methanol
    - Plotting the Methanol Model
  - Growth on Succinate
    - Plotting the Succinate Model
- Concluding Remarks

In this notebook, we give the details of the methods used to fit the partial differential equation model to data obtained from growing *Methylobacterium extorquens* in medium with and without formaldehyde. This description is broadly broken down into three sections:

- The first section describes the PDE model.
- The second section fits the PDE model to data obtained from growing *M. extorquens* under selective conditions: in medium containing moderate concentrations of formaldehyde (HCHO).
- The third section fits the PDE model to data obtained by placing a population selected for high HCHO tolerance in medium without HCHO, with a carbon source of either methanol or succinate.

# The PDE Model

We constructed a population dynamic model to explain the changes observed in HCHO tolerance in *M. extorquens* cells over time. The model consisted of terms that capture growth (on various substrates), death due to elevated HCHO concentrations, unidirectional changes in tolerance phenotype via an advective process, and bidirectional changes in tolerance phenotype via a diffusion process.

The model is given by

\(\partial\_t N(x,t) = r\_c N(x,t) - v~\partial\_xN(x,t) + D~\partial\_{xx}N(x,t) - H(x,F)~N(x,t)\)

where \(N(x,t)\) is the number of cells with tolerance phenotype \(x\) at time \(t\), \(r\_c\) is the growth rate on substrate \(c\), and \(v\) and \(D\) are the advection and diffusion rates, respectively. \(H(x,F)\) is a function that describes death due to HCHO concentration \(F\) and is given by

\(H(x,F) = \alpha (F - b x)\) if \(x<F\)

where \(F\) is the HCHO concentration, \(\alpha\) is the death rate and \(b\) correlates the death rate with increasing sensitivity; note that no death is experienced by cells with tolerance above \(F\). Parameters \(r\_c, D, \alpha,\) and \(b\) are all constrained to be greater than or equal to zero. In the case where \(b = 0\), we have an “absolute” death model where the death rate is equivalent for all cells that have a tolerance below the threshold; otherwise, we consider the model to be a “relative” death model that depends on how far a cell’s tolerance is below the threshold.

In order to numerically solve the above PDE, we must specify both the boundary and initial conditions of the system. The initial conditions are determined using collected data (see below). We chose zero-flux boundary conditions at \(x=0\) and \(x=L\). The lower boundary condition is natural because tolerance cannot fall below 0. The upper boundary condition was chosen because—in the data for any one experiment—tolerance never exceeded a certain level. Mathematically these boundary conditions are expressed as \(\partial\_xN(0,t) = 0\) and \(\partial\_xN(L,t) = 0\).

## Code for the PDE

We use the packages `deSolve` and `ReacTran` to numerically solve the PDE.

```
library(deSolve)
```

```
## Warning: package 'deSolve' was built under R version 3.4.4
```

```
library(ReacTran)
```

```
## Loading required package: rootSolve
```

```
## Loading required package: shape
```

Next, we specify the function used for the PDE. Because we have 4 different versions of the model where either

1. \(v = 0, D = 0\);
2. \(v \ne 0, D = 0\);
3. \(v \ne 0, D \ge 0\);
4. \(v \in \mathbb{R}, D \ge 0\),

we need different solution methods. (1) does not require the use of ReacTran because there is neither advection nor diffusion; (2) requires the use of `advection.1D()`; (3) and (4) can be handled by `tran.1d()`.

```
#note the "opt" flag determines which model is being run
# opt = v gives the advection only model
# opt = d gives a model with diffusion
# all other values give dC = 0
pdefn <- function(time, state, parms, opt="base"){
      with (as.list(parms), {
        dC <- switch(opt,
               v = advection.1D(C=state, flux.up = 0, flux.down = 0, v = V.coef, dx = GR)$dC,
               d = tran.1D(C=state, flux.up = 0, flux.down = 0, D = D.coef, v=V.coef, dx = GR)$dC,
               base = 0)
        Death <- state*alpha*(f-b*x)*(x<f)
        Growth <- state*(rm*(m != 0) + rs*(s != 0))
        return(list(dn = dC + Growth - Death))
      })
    }
```

Most of the parameters of the PDE are to be fit using maximum likelihood, specifically \(v,~ D,~ \alpha,\) and \(b\). We have determined the growth rate \(r\_c\) for methanol and succinate experimentally; these are \(r\_m = 0.195\) for methanol and \(r\_s = 0.267\) \(h^{-1}\) for succinate. The formaldehyde, methanol, and succinate concentration for a given experiment are specified at the time of solution.

## Maximum Likelihood Fitting

In order to fit the variables of interest, we use a maximum likelihood framework. Because of the nature of bacterial growth, it makes sense to measure error on a logarithmic scale. However, due to a number zeroes that naturally occur in the observed data, using a logarithm can be problematic. We therefore used the hyperbolic arcsine function (\(asinh(x) = ln\left(x + \sqrt{1+x^2}\right)\)), which is approximately logarithmic but is defined at \(x=0\).

We assumed that—at the transformation scale—errors should be roughly normally distributed. In other words,

\[z\_i = \mu\_i + \epsilon\] where \[\epsilon \sim \mathcal{N}(0, \sigma\_i)\] and \(z\_i\) is the \(asinh\)-transformed obervation, \(\mu\_i\) is the \(asinh\)-transformed value of PDE solution, and \(\sigma\_i\) is the location specific standard deviation. Under these assumptions, the log-likelihood of our model \(\Theta\) is

\[\mathcal{LL(\Theta)} = -\sum\_i\left(ln(\sigma\_i) + \frac{(x\_i - \mu\_i)^2 }{2 \sigma\_i^2}\right)\] over all observations \(i\). Because this is the standard likelihood formulation used in linear regression, we can use the built-in R algorithms from `lm()` to quickly and easily produce the above likelihood values.

The code to produce the above likelihood calculation for our PDE system is as follows:

```
###Arguments for LL:
### gparms is the set of parameters to be fit (alpha, b, v, D)
### setparms are other parameters necessary to solve the PDE
### IC are the initial conditions for the PDE, specfied at each value of x
### obs are the observed values; should be a data frame having
###     3 columns with these names: Timepoint, mM_HCHO, and CFU
### t0 is the initial time point for the solution
### tmax is the final time point for the solution
### dt is the step size along the time axis
### opt is the opt flag for pdefn (see above)
### fix is a vector of length 4 specifying which parameters are fixed
###     by passing non-NA values in c(alpha = , b = , v = , D = ); e.g.
###     c(0.1, 0, NA, NA) would say fix alpha = 0.1, b = 0 and fit v, D
### retODE is a logical flag that, instead of giving the LL, returns the PDE solution
### retFit is a logical flag that, instead of giving the LL, returns the data frame
###     of fitted values and observed values, NB: retODE takes precedence over this flag
### useDiff is a logical flag that fits the model to "differenced" data value
### trans is a logical flag that, if useDiff = T, determines whether or not the data should 
###     be transformed by asinh() prior to differencing
### smoothing is a logical flag that, if useDiff = T, applies a rule that manipulates the 
###     the data such that the largest possible observations are chosen to give a strictly
###     decreasing measurement of CFU vs tolerance
### forceZeroMin is a logical flag that, if useDiff = T, prevents any CFU values < 0 
require(reshape2)
```

```
## Loading required package: reshape2
```

```
LL <- function(gparms, setparms, IC, obs, t0, tmax, dt = 0.5, opt="base", fix=rep(NA,4), retODE=FALSE, retFit=FALSE, useDiff = FALSE, trans=FALSE, smoothing=FALSE, forceZeroMin=FALSE){

  fixedParms <- !is.na(fix) 
  gparms[fixedParms] <- fix[fixedParms] 
  gparms[3] <- -gparms[3] #invert the sign of the v parameter
  gparms[4] <- abs(gparms[4]) #### NB: D is forced to be positive
  names(gparms) <- c("alpha", "b", "V.coef", "D.coef")
  
  times <- seq(t0,tmax,dt)
  out <- ode.1D(y=IC, times=times, func=pdefn, parms=c(gparms, setparms), dimens = length(x), opt = opt)
  if(retODE) return(out)
  
  # result of the model needs to be aggregated to match the data
  out[,-1] <- t(apply(out[,-1], 1, function(X) rev(cumsum(rev(X))))) 
  out <- as.data.frame(out)
  tmp_model <- melt(out, id.vars = "time", variable.name = "phenotype", value.name = "cfu.m")
  tmp_model$phenotype <- x[tmp_model$phenotype]
  
  #bring in the data, aggregate to unique time x phenotype rows, then get the mean at the asinh-scale,
  data <- obs[,c("Timepoint","mM_HCHO","CFU")]
  colnames(data) <- c( "time", "phenotype", "cfu")
  data <- subset(data, time >= t0)
  data <- aggregate(data[,"cfu", drop=F], by=data[,c("time","phenotype")], FUN=function(X) sinh(mean(asinh(X))))
  
  #merge the model data and the obervations for calculation of the LL
  data <- merge(data, tmp_model, all.x = T)
  data <- data[order(data$time, data$phenotype),]
  
  #do differences instead
  
  if(useDiff){
    theDiff <- function(X, trans=FALSE){
      if(trans) return(sinh(-1*diff(c(asinh(X),0))))
      -1*diff(c(X,0))
    }
    if(smoothing){
      data$cfu <- unlist(aggregate(data[,"cfu",drop=F], by = data[,"time",drop=F], function(X) rev(cummax(rev(X))))$cfu)
    } 
    data$cfu <- unlist(aggregate(data[,"cfu",drop=F], by = data[,"time",drop=F], theDiff, trans=trans)$cfu)
    data$cfu.m <- unlist(aggregate(data[,"cfu.m",drop=F], by = data[,"time",drop=F], theDiff, trans=trans)$cfu.m)
    if(forceZeroMin){
      data$cfu <- pmax(data$cfu,0)
      data$cfu.m <- pmax(data$cfu.m,0)
    }
  }
  
  if(retFit) return(data)
  
  #and finally exclude IC from LL calculation
  data <- data[data$time > t0,]
  
  ll <- logLik(lm(asinh(cfu) ~ offset(asinh(cfu.m)) - 1, data))[1] 
  if(is.finite(ll)) ll else -1e100 #if something happens and ll is infinite, return a very large negative number
}
```

The above function can be passed to an optimizer to find the best parameter values for our model. However, prior to doing so, we need experimental data to fit the PDE system.

# Growth on Methanol and Formaldehyde

The first set of data we will consider comes from cells grown in medium that contained 4 mM HCHO and 15 mM methanol (CH\(\_3\)OH). Subsequently, a time-course of the culture was plated on medium containing varying levels of HCHO to determine the number cells that were tolerant at each test concentration. We need to establish the step size along the \(x\)-axis (tolerance), the initial conditions at each point \(x\), the time span, etc.~for fitting PDE. We choose a relatively small step, 0.01, to ensure accurate solutions.

## The 4mM HCHO Data and Initial Conditions

First, we read in the raw data and set up the \(x\)-grid for the PDE solution based on values in the raw data. We also specify which time point is going to be start of the data to be fit. For this experiment, we chose \(t=2~h\) to be the beginning of the time course (see manuscript for details).

```
CFUcounts_raw <-read.csv('selection_exp_CFU.csv', header=T)

#maxTol = highest tolerance category
maxTol <- max(CFUcounts_raw$mM_HCHO[CFUcounts_raw$CFU > 0]) + 1 #note addition of 1 
dx <- 0.01 #set x grid step size
NClasses <- maxTol/dx #n = number of classes to model 
x <- seq(0, maxTol, by=dx) #x are the points along the x-axis at which we will get a solution
GR <- setup.grid.1D(x.up =-dx/2, x.down = maxTol+dx/2 , N=NClasses+1) # setting up the grid for ReacTran

t0 <- 2 #set intial time
CFUcounts_raw <- CFUcounts_raw[CFUcounts_raw$mM_HCHO < maxTol,] #drop unneeded tolerance levels
```

The next block of code actually calculates the initial condition based upon the value of \(t\_0\) set in the previous block (which was the 2 hour time point for this experiment). The basic strategy here is to fit a spline to the observed data and then interpolate the appropriate \(CFU(x)\) for all \(x\).

```
regData <- CFUcounts_raw[CFUcounts_raw$Timepoint == t0,] #use time=0 for calculation of initial conditions
regData$IHS.CFU <- asinh(regData$CFU) #transform the data to our hyperbolic arcsine scale

#now fit the spline
sf <- splinefun(c(regData$mM_HCHO,6), c(regData$IHS.CFU,0),method="hyman") #added in hard 0 count at 6mM
spline.selection<-data.frame(phenotype=x, spline=sf(x))

#plot the data and spline fit
plot(IHS.CFU ~ mM_HCHO, regData, xlab = "Tolerance (mM HCHO)", ylab = "asinh(CFU)", main = "Spline Fit to Observations")
points(x, sf.p <- sf(x), type="l", col = "green", lwd = 3, lty =2)
```

```
CFU <- sinh(sf.p) #revert to normal scale
CFU <- CFU[1:(which(CFU < 0)[1] - 1)] #discard all values of spline after it crosses the x-axis
dCFU <- diff(-1*CFU) #this simple differencing produces the appropriate cell counts at x, rather than the cumulative counts measured experimentally
mM_HCHO <- x[seq_along(dCFU)] #adjust the x-grid to account for point lost due to differencing

plot(mM_HCHO,dCFU, type="l", log="y", ylab = "Cell abundance (CFU/mL)", xlab = "Tolerance (mM HCHO)", main = "Initial Condition") #look at the IC
```

## Parameter Estimation for the 4mM Growth Experiment

We now have all of the pieces necessary to perform the model fitting. First, let’s try an example call that doesn’t require optimization before proceeding to optimization of the parameters.

```
#We need to pad the IC with appropirate number of 0s in unobserved, high tolerance values
state <- rep(0,NClasses+1)
state[seq_along(dCFU)] <- dCFU

#here are the parameters of the model not being estimated
fixed <-  c(f = 4, m = 15, s = 0, rm = 0.195, rs = 0.267 )

#here is an example call of LL function for alpha = 0.189, b = 0, v = 0, D = 0.
LL(c(0.189, 0, 0, 0), setparms = fixed, IC = state, obs = CFUcounts_raw, t0 = t0, tmax = max(CFUcounts_raw$Timepoint))
```

```
## [1] -98.81412
```

The log likelihood function `LL()` is performing as expected. In order to choose the best model for our data, we will use a likelihood ratio test on the nested models. The simplest model that we will consider is one where there is only death (i.e., \(\alpha > 0\)) and the other 3 parameters (\(b,~v,~D\)) are all zero. We will consider adding a single parameter to the base model. To do this, we maximize the log-likelihood for the 3 potential models:

```
#optimize alpha
subMod <- c(NA, 0, 0, 0)
a.only <- optim(c(0.2,0,0,0), LL, control = list(fnscale = -1), fix = subMod, hessian = T, 
                setparms = fixed, IC = state, obs = CFUcounts_raw, t0 = t0, tmax = max(CFUcounts_raw$Timepoint))
a.only$par[!is.na(subMod)] <- subMod[!is.na(subMod)]

#adjust alpha+b
subMod <- c(NA, NA, 0, 0)
ab <- optim(c(0.2,1,0,0), LL, control = list(fnscale = -1), fix = subMod, hessian = T, 
                setparms = fixed, IC = state, obs = CFUcounts_raw, t0 = t0, tmax = max(CFUcounts_raw$Timepoint))
ab$par[!is.na(subMod)] <- subMod[!is.na(subMod)]

#adjust alpha+D
subMod <- c(NA, 0, 0, NA)
ad <- optim(c(0.2,0,0,0.001), LL, control = list(fnscale = -1), opt = "d", fix = subMod, hessian = T, 
                setparms = fixed, IC = state, obs = CFUcounts_raw, t0 = t0, tmax = max(CFUcounts_raw$Timepoint))
ad$par[!is.na(subMod)] <- subMod[!is.na(subMod)]

#adjust alpha+v... slow
subMod <- c(NA, 0, NA, 0)
av <- optim(c(0.2,0,0.05,0), LL, control = list(fnscale = -1), opt = "v", fix = subMod, hessian = T,
                setparms = fixed, IC = state, obs = CFUcounts_raw, t0 = t0, tmax = max(CFUcounts_raw$Timepoint))
av$par[!is.na(subMod)] <- subMod[!is.na(subMod)]

#create a vector of the LRT (chi-squared) values and sort from best to worst
lrt.2parm <- sort(c("alpha.b" = -2*(a.only$value - ab$value), "alpha.d" = -2*(a.only$value - ad$value), "alpha.v" = -2*(a.only$value - av$value)), decreasing = T)

#put the values in a data frame for display and calculate the p-values
LRT.2parm <- data.frame(Model = names(lrt.2parm), LRT = round(lrt.2parm,3), "p-value" = 1 - pchisq(lrt.2parm,1))
```

Now that we’ve obtained the LL values for the 3 different 2-parameter models, we will choose the one with the highest likelihood as long as it is signficantly better than the base 1-parameter model (again, the \(p\)-value is done using a LRT test).

**LRT values for the possible 2 parameter models**

|  | | |
| Model | LRT | p.value |
|  | | |
| alpha.d | 40.853 | 0 |
| alpha.v | 40.026 | 0 |
| alpha.b | 5.710 | 0.017 |
|  | | |

Looking at the first row of the table we can see that the \(\alpha, D\) model performed the best of any of the 2-parameter models and that this model offered a (highly) significant improvement over the 1-parameter model. Next, we test if a 3-parameter model is supported.

```
###ad + b
subMod <- c(NA, NA, 0, NA)
adb <- optim(c(0.2,0.5,0,0.001), LL, control = list(fnscale = -1), opt = "d", fix = subMod, hessian = T, 
                setparms = fixed, IC = state, obs = CFUcounts_raw, t0 = t0, tmax = max(CFUcounts_raw$Timepoint))
adb$par[!is.na(subMod)] <- subMod[!is.na(subMod)]

###ad + v
subMod <- c(NA, 0, NA, NA)
adv <- optim(c(0.2,0,0.05,0.001), LL, control = list(fnscale = -1), opt = "d", fix = subMod, hessian = T, 
                setparms = fixed, IC = state, obs = CFUcounts_raw, t0 = t0, tmax = max(CFUcounts_raw$Timepoint))
adv$par[!is.na(subMod)] <- subMod[!is.na(subMod)]

lrt.3parm <- sort(c("alpha.d.b" = -2*(ad$value - adb$value), "alpha.d.v" = -2*(ad$value - adv$value)), decreasing = T)
LRT.3parm <- data.frame(Model = names(lrt.3parm), LRT = round(lrt.3parm,3), "p-value" = 1 - pchisq(lrt.3parm,1))
```

**LRT values for the possible 3 parameter models**

|  | | |
| Model | LRT | p.value |
|  | | |
| alpha.d.b | 9.379 | 0.002 |
| alpha.d.v | 1.647 | 0.199 |
|  | | |

The top row of the above table shows that the addition of the \(b\) parameter is the best choice, and that the addition of \(b\) significantly improves the fit of the model. The last step is to see if there is support for the full 4-parameter model.

```
advb <- optim(c(0.2,0.5,0.05,0.001), LL, control = list(fnscale = -1), opt = "d", hessian = T, 
                setparms = fixed, IC = state, obs = CFUcounts_raw, t0 = t0, tmax = max(CFUcounts_raw$Timepoint))
```

The LRT (\(\chi^2 =\) 0.752, \(p =\) 0.3857414) for the 4-parameter is not significant. Therefore, the best model for these data is the 3-parameter model that has diffusion but not advection. We can calculate the standard error of the estimates from the optim output.

**Model Estimates and Standard Errors**

|  | | |
| Parameter | Estimate | SE |
|  | | |
| alpha | 0.214 | 0.024 |
| b | 0.809 | 0.117 |
| D | 0.026 | 0.005 |
|  | | |

## Visualizing the Fitted Model

Now that we have a final model to work with, we would like to visually examine the solution. We first look at the continuous solution.

```
soln <- LL(adb$par, opt = "d", setparms = fixed, IC = state, obs = CFUcounts_raw, 
           t0 = t0, tmax = max(CFUcounts_raw$Timepoint), retODE=T)

logRes <- soln
logRes[,2:ncol(logRes)] <- asinh(logRes[,2:ncol(logRes)])
image(logRes, grid = x, xlab="Time", ylab = "Tolerance", main = "Solution Plotted as asinh(CFU)")
```

```
plot(x, logRes[logRes[,1] == 15,-1], ylab="asinh(CFU)", xlab = "Tolerance", main = "t = 15", type = "l")
```

```
# make dataframes for export later:
soln.df<-data.frame(soln)
colnames(soln.df)<-c('time', x)
logRes.df<-data.frame(logRes)
colnames(logRes.df)<-c('time', x)
```

We can calculate the pseudo-\(R^2\) value of the model by doing the following:

```
fit <- LL(adb$par, opt = "d", setparms = fixed, IC = state, obs = CFUcounts_raw, 
           t0 = t0, tmax = max(CFUcounts_raw$Timepoint), retFit=T)

IHS <- lm(asinh(cfu) ~ offset(asinh(cfu.m)) - 1, fit, subset=time > t0)
pseudoR2 <- 1 - deviance(IHS)/deviance(update(IHS, . ~ -1))
```

The resulting pseudo-\(R^2 =\) 0.97, which indicates that the fit of model was quite good overall. To visualize the fit we can plot the solution together with the observed values.

```
library(ggplot2)
```

```
## Warning: package 'ggplot2' was built under R version 3.4.4
```

```
library(RColorBrewer)
fit <- fit[order(fit$time, fit$phenotype),]

g <- ggplot(fit, aes(x=phenotype, y=cfu, group=as.factor(time), color = as.factor(time)))
g + geom_point()  + geom_line(aes(y=cfu.m)) + theme_bw() + scale_y_log10() + coord_cartesian(ylim = c(1e-2, 5e6)) +
  xlab("Tolerance (mM HCHO)") + ylab("CFU (cumulative)") + 
  scale_color_manual(name="Time", values=brewer.pal(10, 'RdYlBu')[c(2:5,8:10)])
```

```
## Warning: Transformation introduced infinite values in continuous y-axis
```

```
## Warning: Transformation introduced infinite values in continuous y-axis
```

```
#plot it against non-cumulative CFU values
fit$dcfu <- NA
fit$dcfu.m <- NA

for(i in unique(fit$time)){
  subdata <- fit[which(fit$time == i), c("time", "phenotype", "cfu", "cfu.m")]
  fit[which(fit$time == i),] <- cbind(subdata, data.frame(dcfu = -diff(c(rev(cummax(rev(subdata$cfu))),0)), dcfu.m = -diff(c(subdata$cfu.m,0))))  
}

g <- ggplot(fit, aes(x=phenotype, y=dcfu, group=as.factor(time), color = as.factor(time)))
g + geom_point()  + geom_line(aes(y=dcfu.m)) + theme_bw() + scale_y_log10() + coord_cartesian(ylim = c(1e-2, 5e6)) +
  xlab("Tolerance (mM HCHO)") + ylab("CFU") + 
  scale_color_manual(name="Time", values=brewer.pal(10, 'RdYlBu')[c(2:5,8:10)])
```

```
## Warning: Transformation introduced infinite values in continuous y-axis

## Warning: Transformation introduced infinite values in continuous y-axis
```

## Using the “Extended” Initial Condition

Because there is an observational threshold to the phenotypic assay (i.e., the data are censored at certain detection limit), we created an “extended” data set that accounts for the possibility that we may have missed some cells that had higher phenotypes. At the high concentrations where we observed no colonies, we made the most extreme correction by adding 1 whole colony to one of the replicates, to assess whether that added abundance made any difference to model results. We used the following rules to add colonies:

1. If 0 colonies were observed in all technical replicates, add 1 to one of the replicates.
2. Only do (1) if either:
   1. there is an observation of >1 colony at a higher concentration than the one being considered OR
   2. it is the first concentration beyond the last observation of >1 colony.

The extended data set is present in the same spreadsheet that contains the original experimental results, in the column “CFU\_ext”. In the following section, we use the extended data set as the initial conditions for the same analyses as were conducted above.

```
CFUcounts_raw$CFU_orig <- CFUcounts_raw$CFU
CFUcounts_raw$CFU <- CFUcounts_raw$CFU_ext
#maxTol = highest tolerance category
maxTol <- max(CFUcounts_raw$mM_HCHO[CFUcounts_raw$CFU > 0]) + 1 #note addition of 1 
dx <- 0.01 #set x grid step size
NClasses <- maxTol/dx #n = number of classes to model 
x <- seq(0, maxTol, by=dx) #x are the points along the x-axis at which we will get a solution
GR <- setup.grid.1D(x.up =-dx/2, x.down = maxTol+dx/2 , N=NClasses+1) # setting up the grid for ReacTran
t0 <- 2 #set intial time
CFUcounts_raw <- CFUcounts_raw[CFUcounts_raw$mM_HCHO < maxTol,] #drop unneeded tolerance levels
regData_ext <- CFUcounts_raw[CFUcounts_raw$Timepoint == t0,] #use time=0 for calculation of initial conditions
regData_ext$IHS.CFU <- asinh(regData_ext$CFU) #transform the data to our hyperbolic arcsine scale
#now fit the spline
sf <- splinefun(c(regData_ext$mM_HCHO,6), c(regData_ext$IHS.CFU,0),method="hyman") #added in hard 0 count at 6mM
spline.ext<-data.frame(phenotype=x, spline=sf(x))
#plot the data and spline fit
plot(IHS.CFU ~ mM_HCHO, regData_ext, xlab = "Tolerance (mM HCHO)", ylab = "asinh(CFU)", main = "Spline Fit to Extended Observations")
points(x, sf.p <- sf(x), type="l", col = "green", lwd = 3, lty =2)
```

```
CFU <- sinh(sf.p) #revert to normal scale
CFU <- CFU[1:(which(CFU < 0)[1] - 1)] #discard all values of spline after it crosses the x-axis
dCFU <- diff(-1*CFU) #this simple differencing produces the appropirate cell counts at x, rather an a cumulative
mM_HCHO <- x[seq_along(dCFU)] #adjust the x-grid to account for point lost due to differencing
plot(mM_HCHO,dCFU, type="l", log="y", xlab = "mM HCHO", ylab = "Tolerance (mM HCHO)", main = "Extended Initial Condition") #look at the IC
```

```
#We need to pad the IC with appropriate number of 0s in unobserved, high tolerance values
state <- rep(0,NClasses+1)
state[seq_along(dCFU)] <- dCFU
#optimize alpha
subMod <- c(NA, 0, 0, 0)
ext.a.only <- optim(c(0.2,0,0,0), LL, control = list(fnscale = -1), fix = subMod, hessian = T, 
                setparms = fixed, IC = state, obs = CFUcounts_raw, t0 = t0, tmax = max(CFUcounts_raw$Timepoint))
ext.a.only$par[!is.na(subMod)] <- subMod[!is.na(subMod)]
#adjust alpha+b
subMod <- c(NA, NA, 0, 0)
ext.ab <- optim(c(0.2,1,0,0), LL, control = list(fnscale = -1), fix = subMod, hessian = T, 
                setparms = fixed, IC = state, obs = CFUcounts_raw, t0 = t0, tmax = max(CFUcounts_raw$Timepoint))
ext.ab$par[!is.na(subMod)] <- subMod[!is.na(subMod)]
#adjust alpha+D
subMod <- c(NA, 0, 0, NA)
ext.ad <- optim(c(0.2,0,0,0.001), LL, control = list(fnscale = -1), opt = "d", fix = subMod, hessian = T, 
                setparms = fixed, IC = state, obs = CFUcounts_raw, t0 = t0, tmax = max(CFUcounts_raw$Timepoint))
ext.ad$par[!is.na(subMod)] <- subMod[!is.na(subMod)]
#adjust alpha+v... slow
subMod <- c(NA, 0, NA, 0)
ext.av <- optim(c(0.2,0,0.05,0), LL, control = list(fnscale = -1), opt = "v", fix = subMod, hessian = T,
                setparms = fixed, IC = state, obs = CFUcounts_raw, t0 = t0, tmax = max(CFUcounts_raw$Timepoint))
ext.av$par[!is.na(subMod)] <- subMod[!is.na(subMod)]
#create a vector of the LRT (chi-squared) values and sort from best to worst
ext.lrt.2parm <- sort(c("alpha.b" = -2*(ext.a.only$value - ext.ab$value), "alpha.d" = -2*(ext.a.only$value - ext.ad$value), "alpha.v" = -2*(ext.a.only$value - ext.av$value)), decreasing = T)
#put the values in a data frame for display and calculate the p-values
ext.LRT.2parm <- data.frame(Model = names(ext.lrt.2parm), LRT = round(ext.lrt.2parm,3), "p-value" = 1 - pchisq(ext.lrt.2parm,1))
```

```
stargazer::stargazer(ext.LRT.2parm, summary=F, rownames=F, 
                     type="html",title="Model Estimates and Standard Errors")
```

**Model Estimates and Standard Errors**

|  | | |
| Model | LRT | p.value |
|  | | |
| alpha.d | 18.480 | 0.00002 |
| alpha.v | 17.455 | 0.00003 |
| alpha.b | 0.914 | 0.339 |
|  | | |

From the table, we can clearly see that the model that included the diffusion parameter \(D\) outperformed the addition of either advection \(v\), or the more complicated death function \(b\). This is no different than the model without the extended initial conditions. Now on to selection of additional parameters:

```
###ad + b
subMod <- c(NA, NA, 0, NA)
ext.adb <- optim(c(0.2,0.5,0,0.001), LL, control = list(fnscale = -1), opt = "d", fix = subMod, hessian = T, 
                setparms = fixed, IC = state, obs = CFUcounts_raw, t0 = t0, tmax = max(CFUcounts_raw$Timepoint))
ext.adb$par[!is.na(subMod)] <- subMod[!is.na(subMod)]

###ad + v
subMod <- c(NA, 0, NA, NA)
ext.adv <- optim(c(0.2,0,0.05,0.001), LL, control = list(fnscale = -1), opt = "d", fix = subMod, hessian = T, 
                setparms = fixed, IC = state, obs = CFUcounts_raw, t0 = t0, tmax = max(CFUcounts_raw$Timepoint))
ext.adv$par[!is.na(subMod)] <- subMod[!is.na(subMod)]

ext.lrt.3parm <- sort(c("alpha.d.b" = -2*(ext.ad$value - ext.adb$value), "alpha.d.v" = -2*(ext.ad$value - ext.adv$value)), decreasing = T)
ext.LRT.3parm <- data.frame(Model = names(ext.lrt.3parm), LRT = round(ext.lrt.3parm,3), "p-value" = 1 - pchisq(ext.lrt.3parm,1))
```

**LRT values for the possible 3 parameter models**

|  | | |
| Model | LRT | p.value |
|  | | |
| alpha.d.b | 9.379 | 0.002 |
| alpha.d.v | 1.647 | 0.199 |
|  | | |

As with the original IC, the second parameter added to the model using the extended IC is \(b\), leaving us to assess whether addition of \(v\) to the model is supported.

```
ext.advb <- optim(c(0.2,0.5,0.05,0.001), LL, control = list(fnscale = -1), opt = "d", hessian = T, 
                setparms = fixed, IC = state, obs = CFUcounts_raw, t0 = t0, tmax = max(CFUcounts_raw$Timepoint))
```

The LRT (\(\chi^2 =\) 0.029, \(p =\) 0.8653834) for the 4-parameter is not significant in the extended IC case either. Thus, in summary, the use of the extended IC had no effect on the form of the model.

## Comparing The Parameters

While the use of the extended IC did not affect the form of the optimal model, there are likely to be some differences in the parameter values that produce the best fit. Here is the comparison of the two models:

**Model Estimates and Standard Errors**

|  | | |
| Parameter | Estimate | SE |
|  | | |
| alpha | 0.214 | 0.024 |
| b | 0.809 | 0.117 |
| D | 0.026 | 0.005 |
|  | | |

The table shows that using the extended IC led to minor differences between the parameter estimates. In total, it seems as if the detection threshold of empirical methods had little effect on the parameters we inferred.

### Plots of the Extended IC Solution

For comparison purposes, we will plot the solution of the PDE system with our best fit parameters derived from use of the extended initial condtions.

```
soln.ext <- LL(ext.adb$par, opt = "d", setparms = fixed, IC = state, obs = CFUcounts_raw, 
           t0 = t0, tmax = max(CFUcounts_raw$Timepoint), retODE=T)
logRes.ext <- soln.ext
logRes.ext[,2:ncol(logRes.ext)] <- asinh(logRes.ext[,2:ncol(logRes.ext)])
image(logRes.ext, grid = x, xlab="Time", ylab = "Tolerance", main = "Solution Plotted as asinh(CFU), Extended IC")
```

```
plot(x, logRes.ext[logRes.ext[,1] == 15,-1], ylab="asinh(CFU)", xlab = "Tolerance", main = "t = 15", type = "l")
```

```
fit.ext <- LL(ext.adb$par, opt = "d", setparms = fixed, IC = state, obs = CFUcounts_raw, 
           t0 = t0, tmax = max(CFUcounts_raw$Timepoint), retFit=T)
IHS.ext <- lm(asinh(cfu) ~ offset(asinh(cfu.m)) - 1, fit.ext, subset=time > t0)
pseudoR2.ext <- 1 - deviance(IHS.ext)/deviance(update(IHS.ext, . ~ -1))
fit.ext <- fit.ext[order(fit.ext$time, fit.ext$phenotype),]
g <- ggplot(fit.ext, aes(x=phenotype, y=cfu, group=as.factor(time), color = as.factor(time)))
g + geom_point()  + geom_line(aes(y=cfu.m)) + theme_bw() + scale_y_log10() + coord_cartesian(ylim = c(1e-2, 5e6)) +
  xlab("Tolerance (mM HCHO)") + ylab("CFU (cumulative)") + 
  scale_color_manual(name="Time", values=brewer.pal(10, 'RdYlBu')[c(2:5,8:10)])
```

```
## Warning: Transformation introduced infinite values in continuous y-axis
```

```
#plot it against non-cumulative CFU values
fit.ext$dcfu <- NA
fit.ext$dcfu.m <- NA
for(i in unique(fit.ext$time)){
  subdata <- fit.ext[which(fit.ext$time == i), c("time", "phenotype", "cfu", "cfu.m")]
  fit.ext[which(fit.ext$time == i),] <- cbind(subdata, data.frame(dcfu = -diff(c(rev(cummax(rev(subdata$cfu))),0)), dcfu.m = -diff(c(subdata$cfu.m,0))))  
}
g <- ggplot(fit.ext, aes(x=phenotype, y=dcfu, group=as.factor(time), color = as.factor(time)))
g + geom_point()  + geom_line(aes(y=dcfu.m)) + theme_bw() + scale_y_log10() + coord_cartesian(ylim = c(1e-2, 5e6)) +
  xlab("Tolerance (mM HCHO)") + ylab("CFU") + 
  scale_color_manual(name="Time", values=brewer.pal(10, 'RdYlBu')[c(2:5,8:10)])
```

```
## Warning: Transformation introduced infinite values in continuous y-axis
```

```
# make dataframes for export later
soln.ext.df<-data.frame(soln.ext)
colnames(soln.ext.df)<-c('time', x)
logRes.ext.df<-data.frame(logRes.ext)
colnames(logRes.ext.df)<-c('time', x)
results.T16.ext<-data.frame(mM_HCHO=x, asinh_CFU=logRes.ext[logRes.ext[,1] == 16,-1])
```

The resulting pseudo-\(R^2 =\) 0.97 is slightly higher than the model with original IC, indicating that these IC may be a marginally better choice. However, the choice of ICs made little difference to the fit of the PDE model at a qualitative level.

# Growth in the Absence of Formaldehyde

To investigate what happens when HCHO-tolerant cells are grown in the absence of HCHO, we grew cells in media that contained either succinate or methanol as a carbon source, but no HCHO. These cells were assayed at regular intervals to determine their HCHO-tolerance phenotype. Because there is no death due to HCHO, the parameters \(\alpha, ~b = 0\) (i.e., there is potentially only growth, diffusion, and advection in the model). Using the same methods as above, we use the data from this experiment to fit our PDE system. **Because the extended IC performed marginally better in our previous efforts, we will use the extended IC formulation for fitting these models.**

## Growth on Methanol

```
CFUcounts_regrowth <- read.csv("regrowth_exp_CFU.csv", header=T)
CFUcounts_regrowth$Media <- ifelse(grepl("M",CFUcounts_regrowth$Flask), "methanol", "succinate")

CFUcounts_raw <- subset(CFUcounts_regrowth, Media == "methanol")
CFUcounts_raw$CFU_orig <- CFUcounts_raw$CFU
CFUcounts_raw$CFU <- CFUcounts_raw$CFU_ext
#maxTol = highest tolerance category
maxTol <- max(CFUcounts_raw$mM_HCHO[CFUcounts_raw$CFU > 0]) + 2 #note addition of 2 
dx <- 0.01 #set x grid step size
NClasses <- maxTol/dx #n = number of classes to model 
x <- seq(0, maxTol, by=dx) #x are the points along the x-axis at which we will get a solution
GR <- setup.grid.1D(x.up =-dx/2, x.down = maxTol+dx/2 , N=NClasses+1) # setting up the grid for ReacTran
t0 <- 0 #set intial time
CFUcounts_raw <- CFUcounts_raw[CFUcounts_raw$mM_HCHO < maxTol,] #drop unneeded tolerance levels
regData_m <- CFUcounts_raw[CFUcounts_raw$Timepoint == t0,] #use time=0 for calculation of initial conditions
regData_m$IHS.CFU <- asinh(regData_m$CFU) #transform the data to our hyperbolic arcsine scale
#now fit the spline
sf <- splinefun(regData_m$mM_HCHO, regData_m$IHS.CFU,method="hyman") 
spline.m<-data.frame(phenotype=x, spline=sf(x))
#plot the data and spline fit
plot(IHS.CFU ~ mM_HCHO, regData_m, xlab = "Tolerance (mM HCHO)", ylab = "asinh(CFU)", main = "Spline Fit to Methanol")
points(x, sf.p <- sf(x), type="l", col = "green", lwd = 3, lty =2)
```

```
CFU <- sinh(sf.p) #revert to normal scale
CFU <- CFU[1:(which(CFU < 0)[1] - 1)] #discard all values of spline after it crosses the x-axis
dCFU <- diff(-1*CFU) #this simple differencing produces the appropirate cell counts at x, rather an a cumulative
mM_HCHO <- x[seq_along(dCFU)] #adjust the x-grid to account for point lost due to differencing
plot(mM_HCHO,dCFU, type="l", log="y", xlab = "mM HCHO", ylab = "Tolerance (mM HCHO)", main = "Extended Initial Condition for Methanol") #look at the IC
```

```
#We need to pad the IC with appropirate number of 0s in unobserved, high tolerance value
state <- rep(0,NClasses+1)
state[seq_along(dCFU)] <- dCFU
#here are the parameters of the model not being estimated, only methanol is present
fixed <-  c(f = 0, m = 15, s = 0, rm = 0.195, rs = 0.267 )

base.mod <- LL(c(0,0,0,0),setparms = fixed, IC = state, obs = CFUcounts_raw, t0 = t0, tmax = max(CFUcounts_raw$Timepoint))
#optimize D
subMod <- c(0, 0, 0, NA)
m.D <- optim(c(0,0,0,0.001), LL, control = list(fnscale = -1), opt = "d", fix = subMod, hessian = T, 
                setparms = fixed, IC = state, obs = CFUcounts_raw, t0 = t0, tmax = max(CFUcounts_raw$Timepoint))
m.D$par[!is.na(subMod)] <- subMod[!is.na(subMod)]
#optimize D
subMod <- c(0, 0, NA, 0)
m.V <- optim(c(0,0,0.05,0), LL, control = list(fnscale = -1), opt = "v", fix = subMod, hessian = T, 
                setparms = fixed, IC = state, obs = CFUcounts_raw, t0 = t0, tmax = max(CFUcounts_raw$Timepoint))
m.V$par[!is.na(subMod)] <- subMod[!is.na(subMod)]

#create a vector of the LRT (chi-squared) values and sort from best to worst
m.lrt.1parm <- sort(c("D" = -2*(base.mod - m.D$value), "v" = -2*(base.mod - m.V$value)), decreasing = T)
#put the values in a data frame for display and calculate the p-values
m.LRT.1parm <- data.frame(Model = names(m.lrt.1parm), LRT = round(m.lrt.1parm,3), "p-value" = 1 - pchisq(m.lrt.1parm,1))
```

```
stargazer::stargazer(m.LRT.1parm, summary=F, rownames=F, 
                     type="html",title="LRT values for the possible 1 parameter models for Methanol Growth")
```

**LRT values for the possible 1 parameter models for Methanol Growth**

|  | | |
| Model | LRT | p.value |
|  | | |
| v | 7.843 | 0.005 |
| D | 0 | 1 |
|  | | |

It is clear that adding diffusion did not improve model fit (\(p \approx 1\)), but there is support for adding an advection term \(v\) to the model. Now we check to see if diffusion can improve upon the advection model.

```
#optimize D
subMod <- c(0, 0, NA, NA)
m.vD <- optim(c(0,0,0.05,0.001), LL, control = list(fnscale = -1), opt = "d", fix = subMod, hessian = T, 
                setparms = fixed, IC = state, obs = CFUcounts_raw, t0 = t0, tmax = max(CFUcounts_raw$Timepoint))
m.vD$par[!is.na(subMod)] <- subMod[!is.na(subMod)]
```

The LRT (\(\chi^2 =\) -0.008, \(p =\) 1) for the 2-parameter is not significant, suggesting that a model that only includes advection provides the best model. For this model, \(v =\) 0.018 with a standard error of 0.006249.

### Plotting the Methanol Model

```
soln.m <- LL(m.V$par, opt = "v", setparms = fixed, IC = state, obs = CFUcounts_raw, 
           t0 = t0, tmax = max(CFUcounts_raw$Timepoint), retODE=T)
logRes.m <- soln.m
logRes.m[,2:ncol(logRes.m)] <- asinh(logRes.m[,2:ncol(logRes.m)])
image(logRes.m, grid = x, xlab="Time", ylab = "Tolerance", main = "Solution Plotted as asinh(CFU), Methanol")
```

```
fit.m <- LL(m.V$par, opt = "v", setparms = fixed, IC = state, obs = CFUcounts_raw, 
           t0 = t0, tmax = max(CFUcounts_raw$Timepoint), retFit=T)
IHS.m <- lm(asinh(cfu) ~ offset(asinh(cfu.m)) - 1, fit.m, subset=time > t0)
pseudoR2.m <- 1 - deviance(IHS.m)/deviance(update(IHS.m, . ~ -1))
fit.m <- fit.m[order(fit.m$time, fit.m$phenotype),]
g <- ggplot(fit.m, aes(x=phenotype, y=cfu, group=as.factor(time), color = as.factor(time)))
g + geom_point()  + geom_line(aes(y=cfu.m)) + theme_bw() + scale_y_log10() + coord_cartesian(ylim = c(1e-2, 1e8)) +
  xlab("Tolerance (mM HCHO)") + ylab("CFU (cumulative)") + 
  scale_color_manual(name="Time", values=brewer.pal(10, 'RdYlBu')[c(2:5,8:10)])
```

```
## Warning: Transformation introduced infinite values in continuous y-axis

## Warning: Transformation introduced infinite values in continuous y-axis
```

```
#plot it against non-cumulative CFU values
fit.m$dcfu <- NA
fit.m$dcfu.m <- NA
for(i in unique(fit.m$time)){
  subdata <- fit.m[which(fit.m$time == i), c("time", "phenotype", "cfu", "cfu.m")]
  fit.m[which(fit.m$time == i),] <- cbind(subdata, data.frame(dcfu = -diff(c(rev(cummax(rev(subdata$cfu))),0)), dcfu.m = -diff(c(subdata$cfu.m,0))))  
}
g <- ggplot(fit.m, aes(x=phenotype, y=dcfu, group=as.factor(time), color = as.factor(time)))
g + geom_point()  + geom_line(aes(y=dcfu.m)) + theme_bw() + scale_y_log10() + coord_cartesian(ylim = c(1e-2, 1e8)) +
  xlab("Tolerance (mM HCHO)") + ylab("CFU") + 
  scale_color_manual(name="Time", values=brewer.pal(10, 'RdYlBu')[c(2:5,8:10)])
```

```
## Warning: Transformation introduced infinite values in continuous y-axis

## Warning: Transformation introduced infinite values in continuous y-axis
```

```
# make dataframes for export
soln.m.df<-data.frame(soln.m)
colnames(soln.m.df)<-c('time', x)
logRes.m.df<-data.frame(logRes.m)
colnames(logRes.m.df)<-c('time', x)
```

As with the previous models for growth in high formaldehyde conditions, this model does a very good job of fitting the empirical data (\(R^2 =\) 0.97). Interestingly, rather than seeing the bidirectional process being important, we see the unidirectional movement of advection (toward a tolerance of 0) when formaldehyde is absent and growth is on a methanol substrate.

## Growth on Succinate

```
CFUcounts_regrowth <- read.csv("regrowth_exp_CFU.csv", header=T)
CFUcounts_regrowth$Media <- ifelse(grepl("M",CFUcounts_regrowth$Flask), "methanol", "succinate")

CFUcounts_raw <- subset(CFUcounts_regrowth, Media == "succinate")
CFUcounts_raw$CFU_orig <- CFUcounts_raw$CFU
CFUcounts_raw$CFU <- CFUcounts_raw$CFU_ext
#maxTol = highest tolerance category
maxTol <- max(CFUcounts_raw$mM_HCHO[CFUcounts_raw$CFU > 0]) + 2 #note addition of 2 
dx <- 0.01 #set x grid step size
NClasses <- maxTol/dx #n = number of classes to model 
x <- seq(0, maxTol, by=dx) #x are the points along the x-axis at which we will get a solution
GR <- setup.grid.1D(x.up =-dx/2, x.down = maxTol+dx/2 , N=NClasses+1) # setting up the grid for ReacTran
t0 <- 0 #set intial time
CFUcounts_raw <- CFUcounts_raw[CFUcounts_raw$mM_HCHO < maxTol,] #drop unneeded tolerance levels
regData_s <- CFUcounts_raw[CFUcounts_raw$Timepoint == t0,] #use time=0 for calculation of initial conditions
regData_s$IHS.CFU <- asinh(regData_s$CFU) #transform the data to our hyperbolic arcsine scale
#now fit the spline
sf <- splinefun(c(regData_s$mM_HCHO,12), c(regData_s$IHS.CFU,0),method="hyman") 
spline.s<-data.frame(phenotype=x, spline=sf(x))
#plot the data and spline fit
plot(IHS.CFU ~ mM_HCHO, regData_s, xlab = "Tolerance (mM HCHO)", ylab = "asinh(CFU)", main = "Spline Fit to Succinate")
points(x, sf.p <- sf(x), type="l", col = "green", lwd = 3, lty =2)
```

```
CFU <- sinh(sf.p) #revert to normal scale
if(min(CFU) < 0) CFU <- CFU[1:(which(CFU < 0)[1] - 1)] #discard all values of spline after it crosses the x-axis
dCFU <- diff(-1*CFU) #this simple differencing produces the appropirate cell counts at x, rather an a cumulative
mM_HCHO <- x[seq_along(dCFU)] #adjust the x-grid to account for point lost due to differencing
plot(mM_HCHO,dCFU, type="l", log="y", xlab = "mM HCHO", ylab = "Tolerance (mM HCHO)", main = "Extended Initial Condition for Succinate") #look at the IC
```

```
#We need to pad the IC with appropirate number of 0s in unobserved, high tolerance value
state <- rep(0,NClasses+1)
state[seq_along(dCFU)] <- dCFU
#here are the parameters of the model not being estimated, only methanol is present
fixed <-  c(f = 0, m = 0, s = 3.5, rm = 0.195, rs = 0.267 )

base.mod <- LL(c(0,0,0,0),setparms = fixed, IC = state, obs = CFUcounts_raw, t0 = t0, tmax = max(CFUcounts_raw$Timepoint))
#optimize D
subMod <- c(0, 0, 0, NA)
s.D <- optim(c(0,0,0,0.001), LL, control = list(fnscale = -1), opt = "d", fix = subMod, hessian = T, 
                setparms = fixed, IC = state, obs = CFUcounts_raw, t0 = t0, tmax = max(CFUcounts_raw$Timepoint))
s.D$par[!is.na(subMod)] <- subMod[!is.na(subMod)]
#optimize D
subMod <- c(0, 0, NA, 0)
s.V <- optim(c(0,0,0.05,0), LL, control = list(fnscale = -1), opt = "v", fix = subMod, hessian = T, 
                setparms = fixed, IC = state, obs = CFUcounts_raw, t0 = t0, tmax = max(CFUcounts_raw$Timepoint))
s.V$par[!is.na(subMod)] <- subMod[!is.na(subMod)]

#create a vector of the LRT (chi-squared) values and sort from best to worst
s.lrt.1parm <- sort(c("D" = -2*(base.mod - s.D$value), "v" = -2*(base.mod - s.V$value)), decreasing = T)
#put the values in a data frame for display and calculate the p-values
s.LRT.1parm <- data.frame(Model = names(s.lrt.1parm), LRT = round(s.lrt.1parm,3), "p-value" = 1 - pchisq(s.lrt.1parm,1))
```

```
stargazer::stargazer(s.LRT.1parm, summary=F, rownames=F, 
                     type="html",title="LRT values for the possible 1 parameter models for Succinate Growth")
```

**LRT values for the possible 1 parameter models for Succinate Growth**

|  | | |
| Model | LRT | p.value |
|  | | |
| v | 97.197 | 0 |
| D | 0 | 1 |
|  | | |

As with growth on methanol alone, there is strong support for the addition of the advection parameter to model. The final model to test is whether diffusion improves the fit of the model.

```
#optimize D
subMod <- c(0, 0, NA, NA)
s.vD <- optim(c(0,0,0.05,0.001), LL, control = list(fnscale = -1), opt = "d", fix = subMod, hessian = T, 
                setparms = fixed, IC = state, obs = CFUcounts_raw, t0 = t0, tmax = max(CFUcounts_raw$Timepoint))
s.vD$par[!is.na(subMod)] <- subMod[!is.na(subMod)]
```

The LRT (\(\chi^2 =\) 15.918, \(p =\) 6.616203710^{-5}) for the 2-parameter is highly significant, suggesting that a model that includes both advection and diffusion provides the best model. The parameter table for the final model is:

**Model Estimates and Standard Errors on Succinate**

|  | | |
| Parameter | Estimate | SE |
|  | | |
| v | 0.285 | 0.026 |
| D | -0.033 | 0.010 |
|  | | |

### Plotting the Succinate Model

```
soln.s <- LL(s.vD$par, opt = "d", setparms = fixed, IC = state, obs = CFUcounts_raw, 
           t0 = t0, tmax = max(CFUcounts_raw$Timepoint), retODE=T)
logRes.s <- soln.s
logRes.s[,2:ncol(logRes.s)] <- asinh(logRes.s[,2:ncol(logRes.s)])
image(logRes.s, grid = x, xlab="Time", ylab = "Tolerance", main = "Solution Plotted as asinh(CFU), Succinate")
```

```
fit.s <- LL(s.vD$par, opt = "d", setparms = fixed, IC = state, obs = CFUcounts_raw, 
           t0 = t0, tmax = max(CFUcounts_raw$Timepoint), retFit=T)
IHS.s <- lm(asinh(cfu) ~ offset(asinh(cfu.m)) - 1, fit.s, subset=time > t0)
pseudoR2.s <- 1 - deviance(IHS.s)/deviance(update(IHS.s, . ~ -1))
fit.s <- fit.s[order(fit.s$time, fit.s$phenotype),]
g <- ggplot(fit.s, aes(x=phenotype, y=cfu, group=as.factor(time), color = as.factor(time)))
g + geom_point()  + geom_line(aes(y=cfu.m)) + theme_bw() + scale_y_log10() + coord_cartesian(ylim = c(1e-2, 1e9)) +
  xlab("Tolerance (mM HCHO)") + ylab("CFU (cumulative)") + 
  scale_color_manual(name="Time", values=brewer.pal(10, 'RdYlBu')[c(2:5,8:10)])
```

```
## Warning: Transformation introduced infinite values in continuous y-axis
```

```
#plot it against non-cumulative CFU values
fit.s$dcfu <- NA
fit.s$dcfu.m <- NA
for(i in unique(fit.s$time)){
  subdata <- fit.s[which(fit.s$time == i), c("time", "phenotype", "cfu", "cfu.m")]
  fit.s[which(fit.s$time == i),] <- cbind(subdata, data.frame(dcfu = -diff(c(rev(cummax(rev(subdata$cfu))),0)), dcfu.m = -diff(c(subdata$cfu.m,0))))  
}
g <- ggplot(fit.s, aes(x=phenotype, y=dcfu, group=as.factor(time), color = as.factor(time)))
g + geom_point()  + geom_line(aes(y=dcfu.m)) + theme_bw() + scale_y_log10() + coord_cartesian(ylim = c(1e-2, 1e9)) +
  xlab("Tolerance (mM HCHO)") + ylab("CFU") + 
  scale_color_manual(name="Time", values=brewer.pal(10, 'RdYlBu')[c(2:5,8:10)])
```

```
## Warning: Transformation introduced infinite values in continuous y-axis
```

```
# make dataframes for export later
soln.s.df<-data.frame(soln.s)
colnames(soln.s.df)<-c('time', x)
logRes.s.df<-data.frame(logRes.s)
colnames(logRes.s.df)<-c('time', x)
```

As is true for all of the models presented herein, this model does a very good job of fitting the empirical data (\(R^2 =\) 0.97). Interestingly the advection parameter is much stronger (\(v =\) 0.285) in the succinate model than for the methanol model (\(v =\) 0.018), suggesting a stronger reversion toward the low tolerance phenotypes in succinate. It is also interesting that diffusion again plays a role while growing on succinate whereas it did not for growth on methanol.

# Concluding Remarks

Our PDE model performed well in terms of overall fit to all experimental data. Depending on substrate, either advection, diffusion, or both were important to achieving a well-fit model. In the presence of formaldehyde, the death rate of the cells was modulated by the difference between their tolerance phenotype and the level of formaldehyde in the substrate. Future work should investigate mechanisms by which advection and diffusion could be shaping cellular tolerance to formaldehyde.
